# Age-related Vascular Alterations in the Choroid Plexus: Novel Insights from Pathophysiology and Imaging Studies

**DOI:** 10.14336/AD.2025.0735

**Published:** 2026-07-08

**Authors:** Zhe Sun, Chenyang Li, Dominique Leitner, Michelle Wu, Jiangyang Zhang, Thomas Wisniewski, Yulin Ge

**Affiliations:** ^1^Department of Radiology, NYU Grossman School of Medicine, New York, NY, USA.; ^2^Vilcek Institute of Graduate Medical Sciences at NYU Grossman School of Medicine, New York, NY, USA.; ^3^Depeatment of Neurology, NYU Grossman School of Medicine, New York, NY, USA

**Keywords:** choroid plexus, magnetic resonance imaging, vascular degeneration, aging

## Abstract

The choroid plexus (ChP), a highly vascularized brain structure responsible for cerebrospinal fluid (CSF) production, undergoes significant age-related changes that may contribute to neurodegenerative diseases involving disrupted immune regulation, fluid homeostasis and waste clearance. Compared to other brain regions, vascular research on the ChP remains limited despite its critical role as a central interface between the blood and CSF. This review focuses on age-related vascular and structural alterations in the ChP from both histopathological and neuroimaging perspectives, and explores their impact on CSF dynamics, immune regulation, and the integrity of the blood-CSF barrier (BCSFB). Rather than shrinking, the aging ChP often enlarges due to dystrophic changes, as shown in volumetric MRI studies. Histological studies reveal epithelial degeneration, basement membrane thickening, and stromal fibrosis in the normal aging process. In dementia such as Alzheimer’s disease (AD), proteomic studies have identified upregulation of AD- and immune-related proteins, along with downregulation of proteins linked to CSF clearance and metabolic support. Emerging high-resolution contrast-enhanced MRI techniques now allow in vivo visualization of microvascular changes within the ChP, shedding light on its normal and abnormal aging processes. Understanding these alterations is critical, as they may influence the onset and progression of various neurological diseases such as AD, Parkinson’s disease (PD), normal pressure hydrocephalus, and amyotrophic lateral sclerosis (ALS). The recent advancements and challenges described in this study underscore the need for deeper investigation into ChP aging to inform future diagnostic and therapeutic strategies of neurodegenerative diseases.

## 1. Introduction

The choroid plexus (ChP), situated along the ventricular floor of the brain, is a highly vascularized structure that serves as a critical interface between the blood and cerebrospinal fluid (CSF). It fulfills vital functions in CSF production and circulation, including the involvement of the recently discovered glymphatic system for waste clearance, which is associated with cognitive decline in aging populations. Age-related morphological and functional changes in the ChP have been documented, with implications for neurodegenerative diseases such as Alzheimer's disease (AD), Parkinson's disease (PD), cerebrovascular accidents, and hydrocephalus [1-3]. However, a comprehensive review focusing on vascular degenerative changes of the ChP regarding their histopathological alterations and in vivo imaging findings remains lacking.

ChP blood flow, which is essential for CSF production, is modulated and accompanied by arterial pulse wave, impacting both bulk CSF flow and interstitial circulation via CSF production and circulation. Histopathologically, aging induces vascular degeneration in the ChP, evident at both macroscopic and microscopic levels. Macroscopic changes include vascular wall remodeling, elastin fiber fragmentation, and collagen deposition due to mechanical stress. Microscopically, studies [4-6] have identified capillary wall thickening, endothelial dysfunction, and reduced vascular density, often associated with age-related chronic inflammation. These alterations can impair ChP functions such as CSF secretion, waste filtration, and the regulation of inflammatory mediators [7].

Historically, insights into ChP changes have been primarily derived from histological studies due to the ChP's small size, intraventricular location, and the limitations of noninvasive imaging techniques. As a result, in vivo evidence elucidating the ChP's role in neurological disorders has remained limited. Recent advances in imaging technologies have begun to overcome these challenges, allowing the detection of ChP alterations associated with aging and neurodegenerative diseases [8-10]. However, to our knowledge, imaging studies investigating the ChP and its role in various diseases are still in the early stages of development. Most studies have focused on volumetric changes, which lack diagnostic specificity. Imaging metrics such as vascular perfusion, microstructural diffusion, and BCSFB permeability may offer improved sensitivity and specificity, particularly in conditions like AD [8], and may detect changes that precede volumetric alterations [11]. Additionally, further post-mortem histopathological studies are necessary to validate imaging findings and elucidate disease-specific changes in the ChP.

This review aims to explore vascular changes in the ChP related to aging and dementia, integrating perspectives from histopathology and neuroimaging. A deeper understanding of these vascular alterations may aid in identifying reliable biomarkers and developing therapeutic strategies to mitigate cognitive decline and other neurological disorders associated with aging.

**Figure 1.**
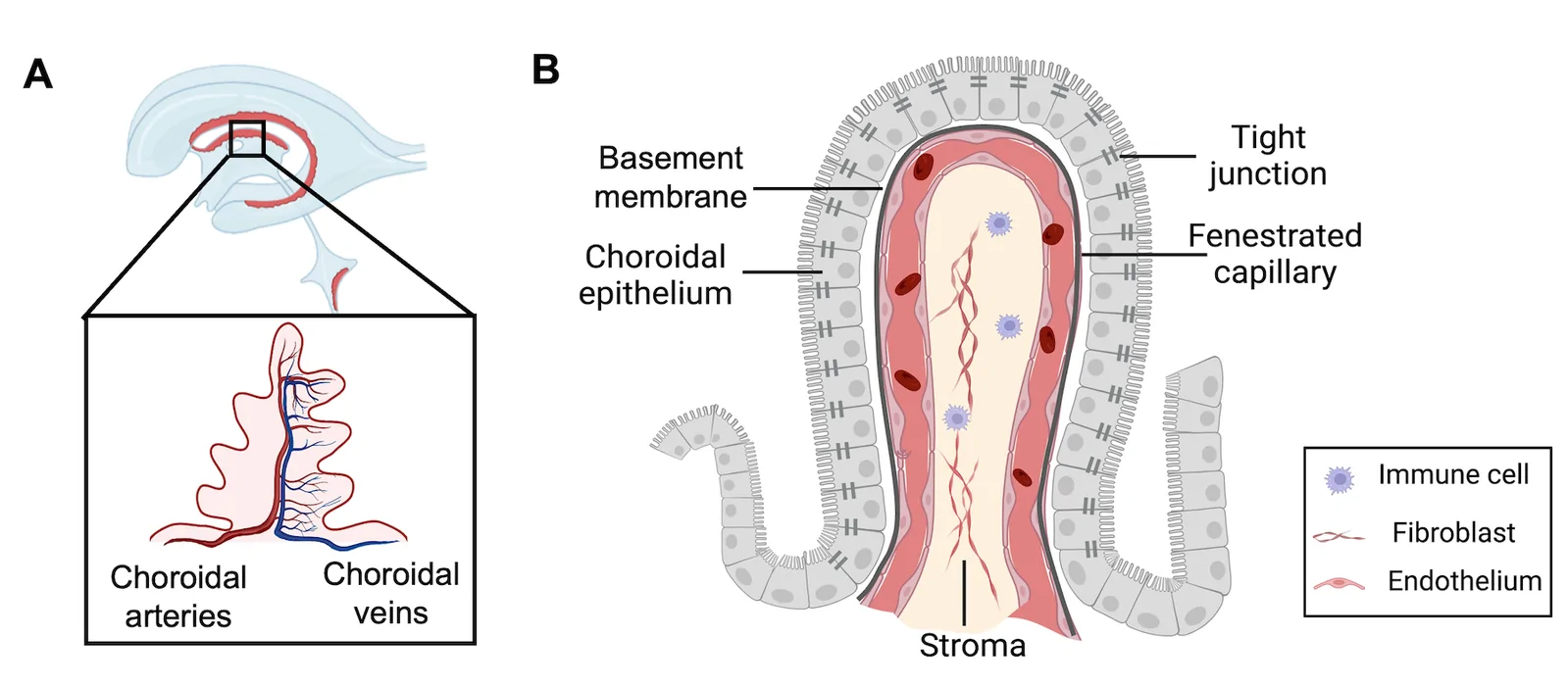
**Schematic overview of the choroid plexus (ChP) anatomy**. (**A**) The ChP is a highly vascularized, secretory structure located within the ventricular system, characterized by numerous villi enriched choroidal arteries and veins. (**B**) Each villus consists of three main components: a layer of choroidal epithelial cells interconnected by tight junctions, stromal tissue, and fenestrated capillaries.

## 2. Anatomy and functions of ChP

### 2.1 Structural and vascular anatomy of the ChP

The ChP is a densely vascularized structure with numerous microvilli projecting into the ventricles, enhancing CSF transport and secretion [12]. The ultrastructural details of the ChP and its vasculature are illustrated in Figure 1. Each choroidal villus is enveloped by a monolayer of cuboidal epithelial cells and fenestrated capillaries. These epithelial cells, anchored on a basement membrane, are primarily responsible for CSF production [12, 13]. Tight junctions between the epithelial cells establish the blood-CSF barrier (BCSFB), effectively preventing unregulated molecular passage from the blood into the CSF [12, 13]. Fenestrated capillaries permit the unrestricted diffusion of small molecules, ions, and water, facilitating efficient water transport from the blood to the epithelial cells and thereby enhancing CSF production. These capillaries are separated from the ChP epithelial cells by connective tissue known as the stroma, which originates from the mesodermal layer [14]. Composed of fibroblasts, collagen fibrils, and immune cells, the ChP stroma provides essential structural support and anchorage for the epithelial cells and capillaries [15].

**Figure 2.**
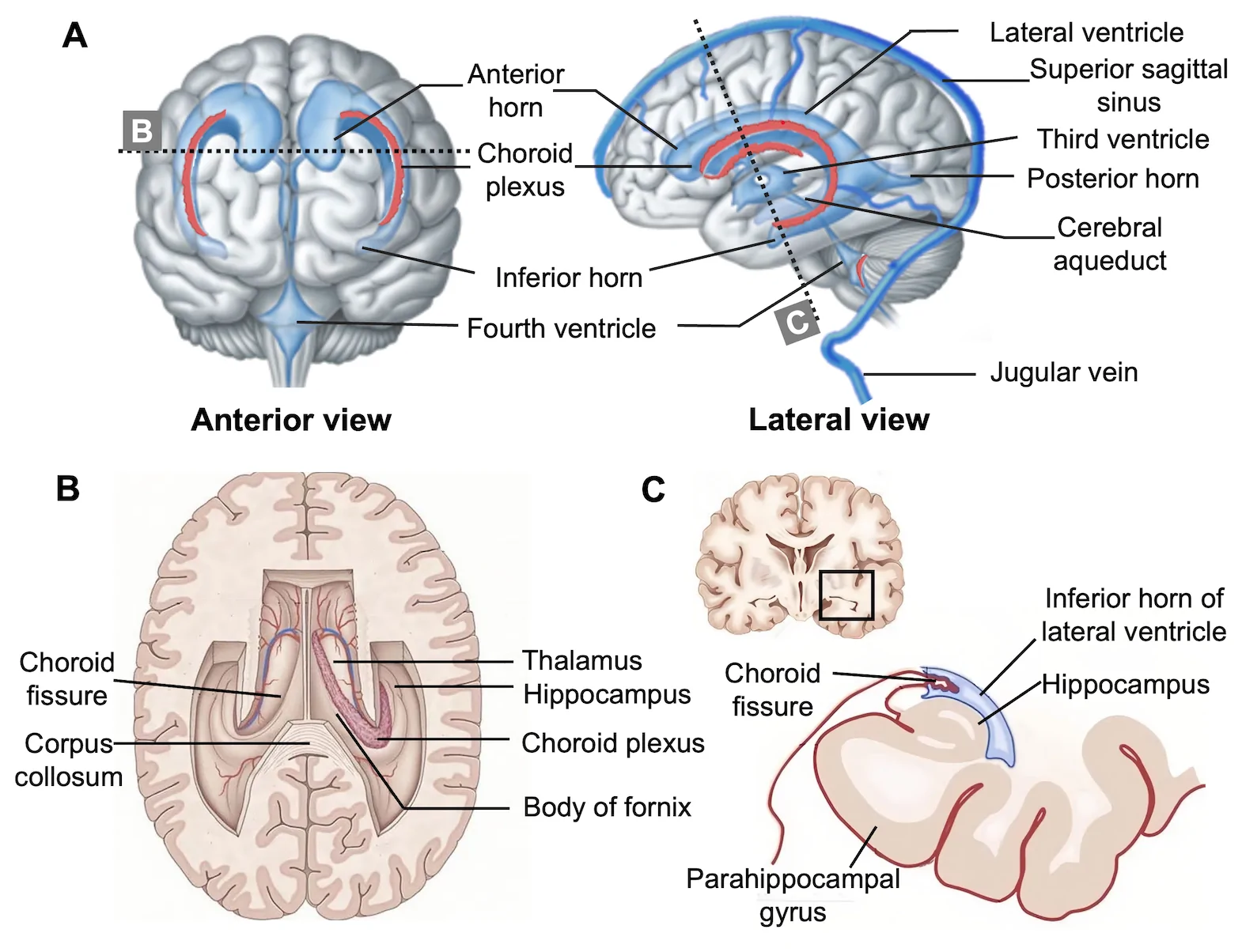
**Illustration of the ChP in the ventricles from different views**. (**A**) Anterior and lateral views of the ventricles, with dissection planes indicated by black dashed lines. (**B**) Cross-sectional view of the lateral ventricle. (**C**) Sectional view through the inferior horn of the lateral ventricle.

**Figure 3.**
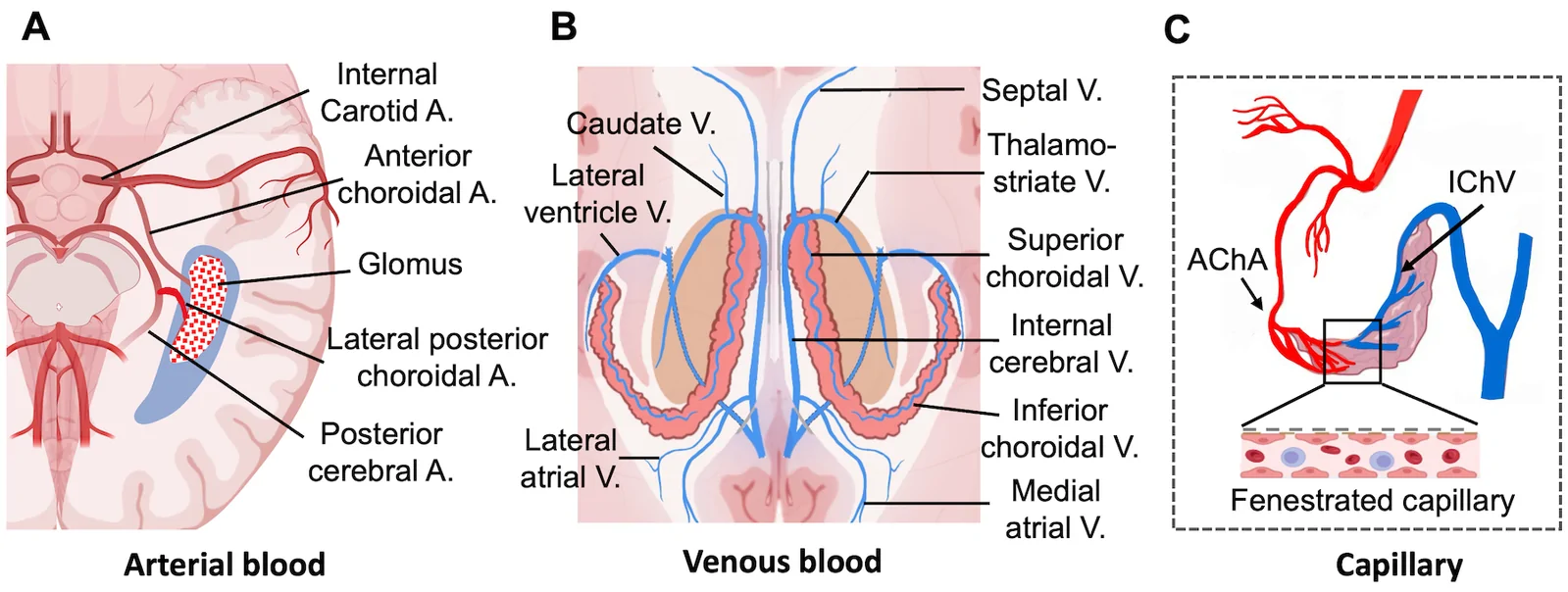
**Schematic overview of the ChP vasculature**. (**A**) The main arterial blood supply of the ChP originates from the anterior choroidal artery (AChA) and the lateral posterior choroidal artery (LPChA), which arise from the internal carotid artery (ICA) and posterior cerebral artery (PCA), respectively. (**B**) The superior and inferior choroidal veins drain the venous blood from the ChP. The superior choroidal vein (SChV) converges into the thalamo-striate vein, and the inferior choroidal vein (IChV) converges into the internal cerebral vein (ICV). (**C**) Within the ChP, fenestrated arterioles and venules connect and form a rich capillary network, i.e., ChP glomus.

The ChP primarily receives its blood supply from two arteries: the anterior choroidal artery (AChA) and the lateral posterior choroidal artery (LPChA). To better illustrate the course of vessels and the spatial relationships of key structures, the ventricular system and major anatomical landmarks associated with the choroid plexus (ChP) are shown in Figure 2. The AChA typically originates from the internal carotid artery (ICA), while the LPChA arises from the posterior cerebral artery (PCA) (Fig. 3A). As depicted in Figure 2A, the AChA traverses the choroid fissure and extends into the inferior horn of the LV, where it bifurcates into medial and lateral branches. The medial branch follows the ventricular margin, whereas the lateral branch aligns with the free margin and gives rise to numerous tributaries. The AChA, with an approximate diameter of 0.6±0.16 mm [16], along with its perforating branches, supplies various subcortical structures, including the amygdala, hippocampus, and substantia nigra. Due to its extensive distribution, ischemic or hemorrhagic events involving the AChA can result in severe complications and potentially fatal outcomes. The ChP located within the third ventricle is supplied by the medial posterior choroidal artery (MPChA), a branch of the PCA [16, 17].

The venous drainage of the ChP is primarily facilitated by the superior choroidal vein (SChV) and the inferior choroidal vein (IChV). The SChV courses along the roof of the lateral ventricle (LV), collecting venous blood from the anterior portion of the ChP. It subsequently merges with the thalamostriate vein to form the internal cerebral vein (ICV) near the foramen of Monro. The IChV drains the inferior segment of the ChP and empties into the basal vein (of Rosenthal) [18]. These choroidal veins also receive venous drainage from adjacent structures, including the hippocampus and deep temporal white matter (WM) (Fig. 3B). Proximal to the IChV, the medial atrial vein (MAV) and lateral atrial vein (LAV) primarily facilitate drainage from the ventricular walls. Ultimately, the venous blood from the ChP converges into the ICVs, which unite with the basal veins to form the great cerebral vein (of Galen). This vein then drains into the straight sinus, which subsequently empties into the superior sagittal sinus (SSS) and exits the skull via the internal jugular veins [19].

**Figure 4.**
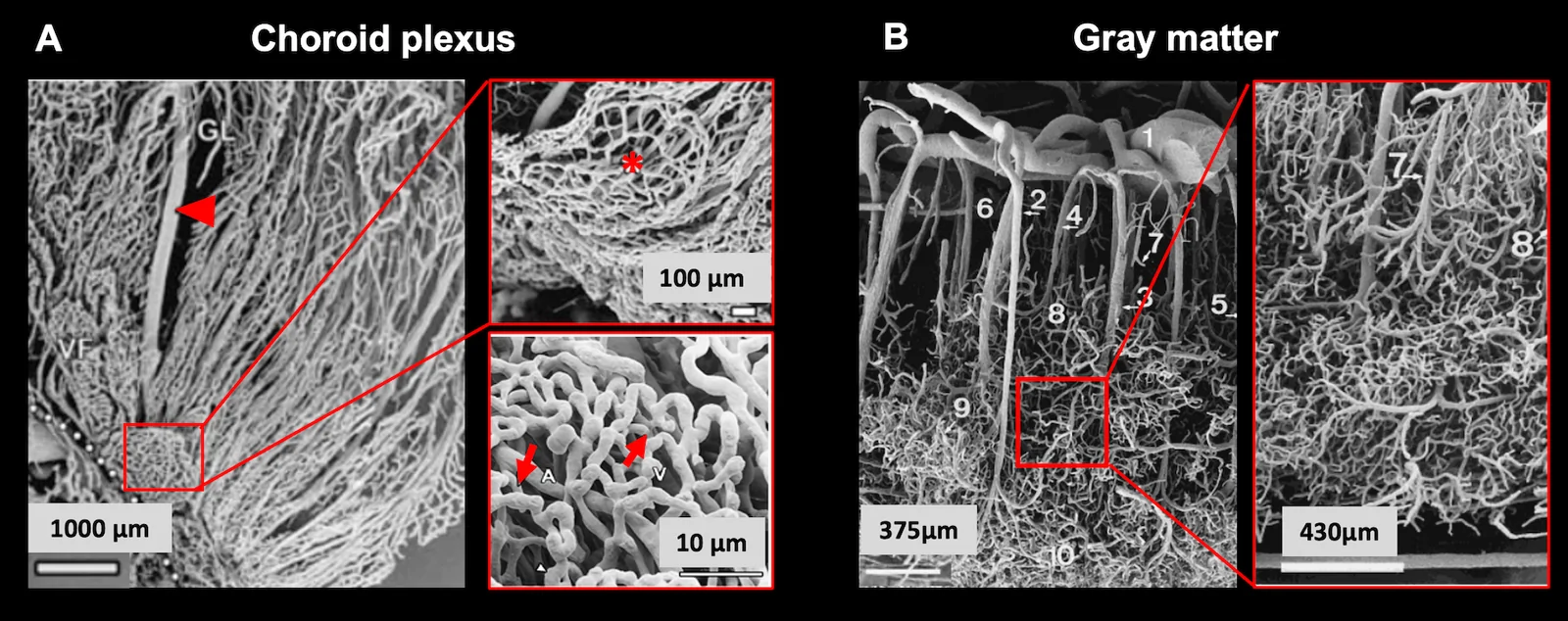
**Electron microscopy of vascular configuration in ChP and gray matter (GM)**. (**A**) The ChP contains a capillary network, forming tubular plexuses that tangle with the supplying vessels (red arrowhead). It has a large number of vascular anastomoses (asterisk) with direct arteriolar-venular (A-V) connections (red arrows). (**B**) In contrast, the cortical capillary network within the GM with smaller diameters have less anastomoses and lack A-V connections. Adapted from Ref [6, 22].

As shown in Figure 3C, the macrovasculature of the ChP includes key vessels such as the LPChA and IChV. These vessels are enveloped by a dense, mesh-like network of capillaries rich in anastomoses, forming the ChP glomus [20] — a vascular configuration distinct from that of other brain tissue structures. This unique vascular configuration, characterized by high tortuosity, rich vascularity, and fenestrated capillaries, results from extensive embryonic angiogenesis and expansion during brain development [18, 21]. Electron microscopy reveals that the ChP vasculature includes frequent vascular anastomoses and short arteriolar-venular (A-V) connections, with capillary diameters averaging around 15 μm [6] (Fig. 4A). In contrast, the capillary network in the gray matter (GM) cortex consists of vessels typically ≤7 μm in diameter and exhibits fewer anastomoses and A-V connections [22] (Fig. 4B). This distinction highlights the structural and functional differences between the ChP and other brain tissues, with the ChP demonstrating a blood flow rate approximately 1.6 times greater than that of the GM [23] and 2.5 times greater than in the WM. This high blood flow in the ChP with relatively low cellular metabolic demand mainly contributes to the ChP perfusion and CSF production.

### 2.2. Functions of ChP

The ChP primarily functions in the production of CSF, barrier maintenance, waste clearance, and immuno-regulation. In this review, we aim to explore these ChP functions, particularly its involvement in age-related neurodegenerative diseases.

#### 2.2.1 CSF production and dynamics

Under normal physiological conditions, the ChP epithelial cells are primarily responsible for CSF production, generating more than 50% of the total CSF at a rate of approximately 400 to 600 mL per day in young adults [24, 25]. The remaining CSF is derived from interstitial fluid (ISF) produced by the blood-brain barrier (BBB) and the ependymal cells lining the ventricles [24, 26].

CSF production in the ChP involves two main steps:

- *Passive Filtration:* Plasma is passively filtered from choroidal capillaries into the choroidal interstitial compartment across the basement membrane, driven by a pressure gradient. The ChP capillaries are leaky with 60 to 80 nm fenestrations [13], allowing small molecules to pass through and facilitating the rapid delivery of water to epithelial cells for CSF production [27, 28].
- *Active Transport:* Fluid is actively transported from the interstitial compartment to the ventricular lumen through the choroidal epithelium, facilitated by aquaporins (AQPs) and membrane carrier proteins. Among these transporters, AQP1, expressed in the apical membrane of ChP epithelial cells, plays a predominant role, contributing 20-25% of the CSF produced by the ChP [29].

The average adult CSF volume in the brain is approximately 150mL, with about 125mL located in the subarachnoid space and 25mL within the ventricles. This total volume is renewed roughly four times per day, a process essential for waste removal [27]. Previously, CSF drainage and clearance were thought to occur primarily through absorption into the venous system via arachnoid granulations. These small protrusions of the arachnoid mater extend into the dural venous sinuses and facilitate the transfer of CSF from the subarachnoid space into the bloodstream. Beyond this classic pathway, various transmembrane proteins expressed in the ChP epithelium enable selective transport of metabolites and waste solutes between the blood and CSF. For instance, multidrug resistance-associated proteins (MRPs), such as MRP1 encoded by the *ABCC1* gene, are expressed on epithelial cells and can transport metabolic waste products and harmful molecules, including heavy metal ions. Low-density lipoprotein receptor-related proteins (LRPs) localized on the apical side of epithelial cells, can clear peptides and peptide fragments, such as amyloid-beta (Aβ), from the CSF [30]. These transporter proteins are crucial for maintaining metabolic homeostasis in the CSF.

#### 2.2.2 Blood-CSF barrier function

**Figure 5.**
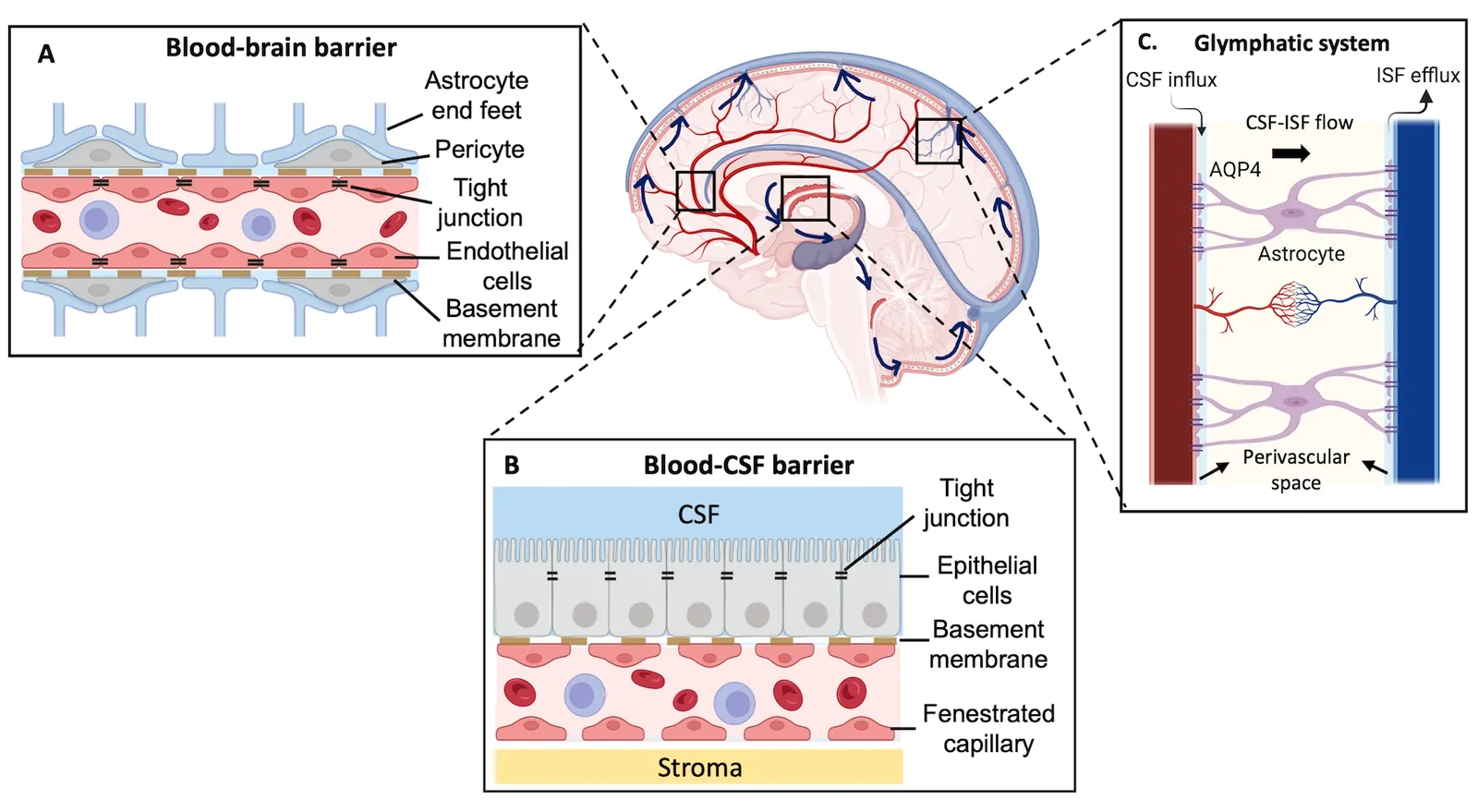
**Blood-brain barrier (BBB), blood-CSF barrier (BCSFB), and glymphatic system**. (**A**) The BBB is formed by the endothelial cells connected by tight junctions, pericytes, and astrocyte end feet, which separates blood circulation from the brain tissue; (B) The BCSFB is composed of ChP epithelial cells interconnected by tight junctions, basement membrane between vessels and epithelial cells, and fenestrated capillary. The tight junctions located in the ChP epithelial cells are different from those of the BBB, which are found on the endothelial cells of vascular capillaries. BCSFB creates a selective permeability to molecules, ions, and cells, allowing for the precise control of the CSF composition and homeostasis; (C) The glymphatic system involves the exchange of CSF with interstitial fluid in the brain. It relies on connective flow facilitated by CSF production, astrocytic water channels and perivascular spaces for waste clearance.

The central nervous system (CNS) is effectively shielded from whole body systemic fluctuations by two primary barriers: the BBB and the BCSFB. The BBB is a sophisticated system consisting of capillary endothelial cells, pericytes embedded within the basement membrane, and astrocytic end feet (Fig. 5A). This barrier impedes the free paracellular passage of water-soluble molecules through tight junctions between the endothelial cells of capillaries. The combination of tight junctions and the enveloped vascular endothelium contributes to its limited permeability, thereby ensuring the protection of brain tissues such as GM and WM.

Unlike the BBB, the BCSFB separates blood in the ChP from CSF through a monolayer of choroidal epithelial cells interconnected by apical tight junctions (Fig. 5B). Due to the leaky structure of choroidal capillaries, they are permeable to nearly all proteins [31]. However, the tight junctions at the ChP epithelial cells could prevent the free passage of large molecules, allowing the BCSFB to regulate the transport of harmful substances and maintain intracranial homeostasis through barrier proteins and tight junctions [32]. For example, Aβ and tau can pass through the fenestrations but are subject to regulation by the BCSFB. In the context of AD, levels of Aβ1-40 peptides, total tau (t-tau) and phosphorylated tau (p-Tau) levels are notably elevated in the CSF [33].

While the degree of sealing in the endothelium's paracellular pathway remains uncertain, fenestrated choroidal capillary loops may allow the diffusion of molecules up to approximately 800 kDa, including most gadolinium-based contrast agents (GBCAs), into the stroma [34]. The differences between the BBB and BCSFB could affect the choice of kinetic model when evaluating permeability using Gd-enhanced MRI.

#### 2.2.3 Role of the ChP in the glymphatic system

Beyond the role of CSF production and circulation driven by the ChP, recent studies have demonstrated that CSF and waste solutes circulate through the para-vascular spaces of blood vessels penetrating the brain parenchyma and play a role in waste clearance of the brain [35]. This process involves the exchange of para-arterial CSF and para-venous ISF, facilitated by glial AQP4 channels located on astrocytic endfeet [36]. The glymphatic system provides a brain-wide clearance pathway of neurotoxic substances, such as Aβ.

Given the primary role of the ChP in CSF production and ion regulation, it creates pressure that drives the glymphatic flux and enables glymphatic CSF-ISF exchange (Fig. 5C). It has been suggested that CSF production rates align with glymphatic clearance rates, with both peaking during sleep [37, 38]. During sleep or anesthesia, there is a notable expansion of the brain interstitial space volume compared to the awake state, which reduces overall resistance to para-vascular inflow, consequently accelerating the CSF-ISF exchange and convective transportation of waste solutes [37]. Under normal conditions, the ChP may increase CSF production to expedite waste clearance in response to increased concentrations of waste substances, given its critical role in immune surveillance and response [39]. Future investigations are necessary to elucidate the interactions between ChP function and the glymphatic system under various physiological regulation, such as respiration, circadian rhythm, and postural shift.

#### 2.2.4 Immune surveillance role

In healthy individuals, the ChP and arachnoid matter function as neuroimmune gateways for CNS immunosurveillance, as immune system cells are detectable in the CSF rather than within CNS parenchyma [40, 41]. Various immune cells, including macrophages, neutrophils, dendritic cells, B cells, and T cells, are associated with the ChP, residing in the stromal tissue and adhering to the CSF-facing membrane of the epithelial cells [40]. The ChP's high blood flow rate, anastomosed capillary network, and fenestrated wall structure allow for extensive exposure of circulating immune cells to the ChP [42]. These immune cells transverse the ChP by migrating from the blood through the permeable capillaries into the stromal space, and then cross the basolateral surface of the ChP epithelium to enter the ventricles [13].

Immunoinflammatory cells such as T cells, monocytes, and dendritic cells found in CSF are more specific and sensitive indicators of CNS pathology than their counterparts in blood. CSF typically presents a relatively higher concentration of these cells compared to their composition in the blood. The chemokine composition can undergo significant changes under pathological conditions, even though the exact mechanisms are not fully understood [32, 42]. An overload of immune cells and inflammatory cytokines can stimulate the stromal connective tissue through the extracellular matrix, leading to ChP enlargement. This enlargement is commonly observed in certain neuroinflammatory diseases, such as multiple sclerosis, as evidenced in structural MRI.

Therefore, the ChP plays a critical role in CSF production, waste clearance, and immunoregulation, all of which are associated with neurological pathologies. Its dense vascularization and capillary wall structure allows for abundant hemodynamics, making it distinct from other brain tissues. However, it remains challenging for conventional MRI techniques to acquire detailed anatomical structures *in vivo*, track the rapid outflow, and assess the BCSFB permeability and function. These challenges arise due to the small size, limited imaging resolution and contrast, and its immersion in CSF [23, 43, 44].

## 3. Age-related pathophysiological changes of ChP

The ChP undergoes both structural and functional modifications with aging, including epithelial atrophy, loss of tight junction integrity, and decreased CSF production and waste clearance. These alterations can result in the accumulation of neurotoxic substances and contribute to cognitive dysfunction and various brain deficits. Therefore, age-related changes in the ChP, as detected through neuroimaging, have the potential to serve as biomarkers for early diagnosis and monitoring of several age-related neurodegenerative diseases.

### 3.1 Morphological changes

**Figure 6.**
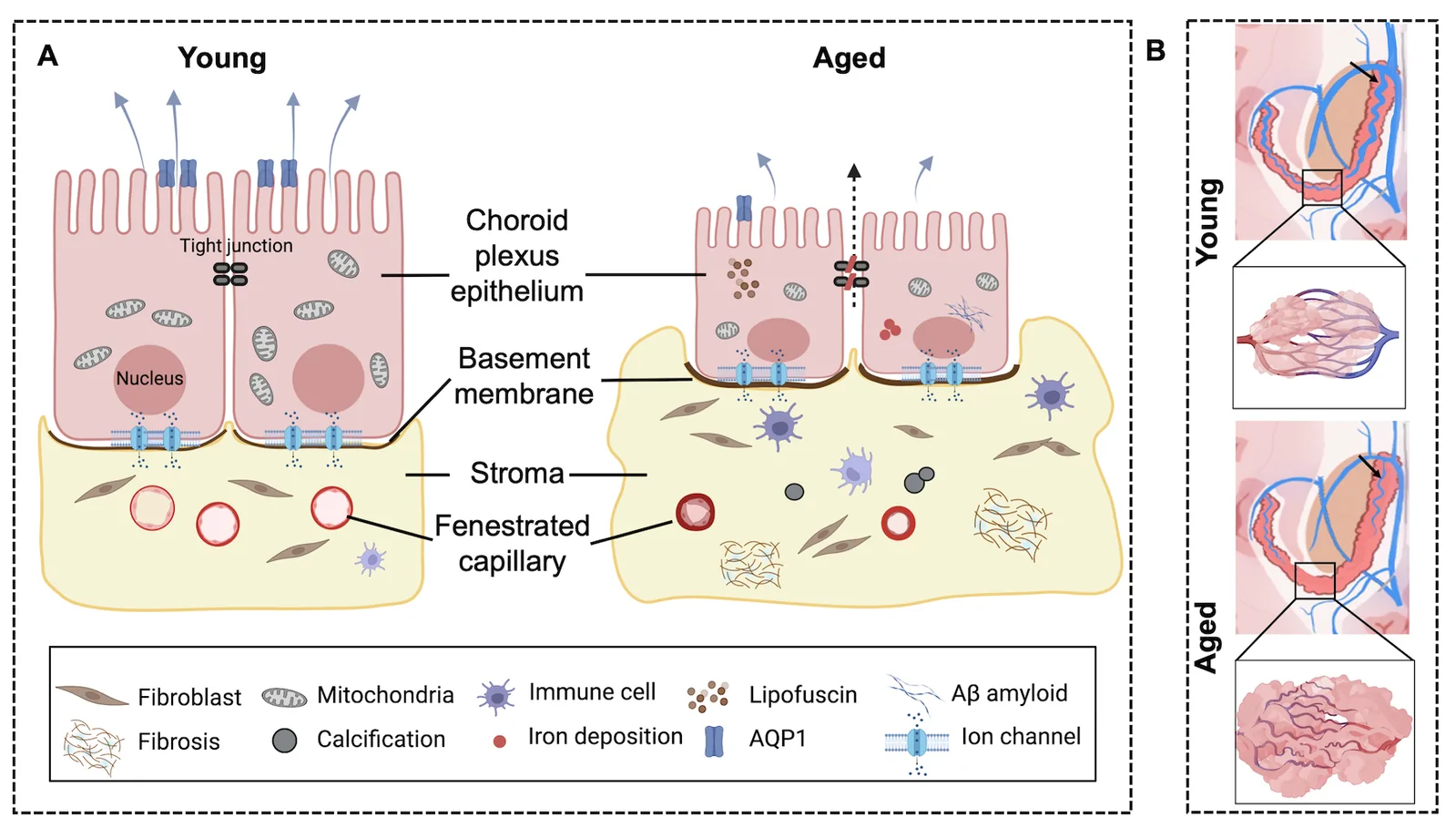
**Age-related morphological alterations in the ChP**. (**A**) The ultrastructure of ChP undergoes morphological changes including shortened epithelial cells and microvilli and expanded stromal tissue et al. (**B**) The vasculature of the ChP exhibits age-related atrophy and loss.

With aging, the ChP commonly enlarges, as demonstrated by histopathology and imaging studies. This enlargement may result from stromal fibrosis, hypertrophy, and chronic low-grade inflammation that stimulates the production of growth factors such as insulin, insulin-like growth factor (IGF)-1, and IGF-2 to support epithelial health [28, 32], leading to stromal cell proliferation and increased volume [45]. Additionally, the accumulation of waste products, calcifications, lipofuscin, and basement membrane thickening also contribute to this enlargement [46, 47] (Fig. 6A). Despite the increased size, ChP epithelial cells become flatter, with a reduced height of approximately 11%) [5], irregular nuclei, shortened microvilli, and fewer transporter proteins, which collectively result in a negative impact on CSF production [48].

Recent studies have identified fibroblasts in the ChP stroma, alongside immune cells, pericytes, and vascular smooth muscle cells [13, 40, 49]. These fibroblasts likely secrete extracellular matrix components and provide structural support. Aging and conditions such as AD can exacerbate fibrosis, calcification, and vascular degeneration in the ChP [50]. Although the specific functions of ChP fibroblasts remain unclear, they may respond to changes in blood flow and tissue stiffness, potentially aiding in vascular remodeling [51]. An increase in the volume of the ChP, particularly within the stroma, might be a compensatory mechanism to counterbalance the age-related decline in ChP blood flow and CSF turnover [26].

Currently, T1-weighted (T1w) structural MRI is commonly used to visualize ChP morphology. Studies consistently show enlargement of the ChP and lateral ventricles with aging and in certain neurological diseases [9, 52, 53]. However, accurately quantifying ChP volume is challenging due to the unclear boundary between the ChP and CSF caused by the partial volume effect (PVE). Johns et al. have recently utilized a CUBIC optical tissue clearing technique to examine interactions within the ChP stroma, providing insights into the role of fibroblasts in ChP enlargement and immune filtration during aging [54].

### 3.2 Vasculature changes

Both macro-vessels (arteries and veins) and micro-vessels (capillaries) within the ChP undergo age-related changes (Fig. 6B). The ChP-supplying arteries, such as AChA and LPChA, typically measure between 0.7-1.2 mm in diameter [55]. With aging, small arteries undergo inward hypertrophic remodeling, characterized by reduced lumen diameter and increased wall thickness. Simultaneously, chronic inflammation triggered by barrier dysfunction promotes fibrosis and thickening of the vessel basement membranes. Capillary density also declines with age, resulting in a fragmented or atrophied microvascular networks [52], which impairs blood flow and adversely affects CSF production.

Repetitive pulsatile blood flow imposes mechanical stress on the vessel walls, which can activate inflammatory signaling pathways within endothelial cells. This prompts the release of growth factors, driving vascular wall remodeling and extracellular matrix alterations [56]. Such remodeling frequently manifests as tortuous or twisting vessel geometry, a common characteristic observed across cerebral arteries of varying sizes [57]. These tortuous changes may compromise blood and oxygen supply, potentially leading to infarction. However, due to the rich vascular network of villous tributaries and abundant anastomoses within the ChP, infarction in this tissue remains rare.

**Figure 7.**
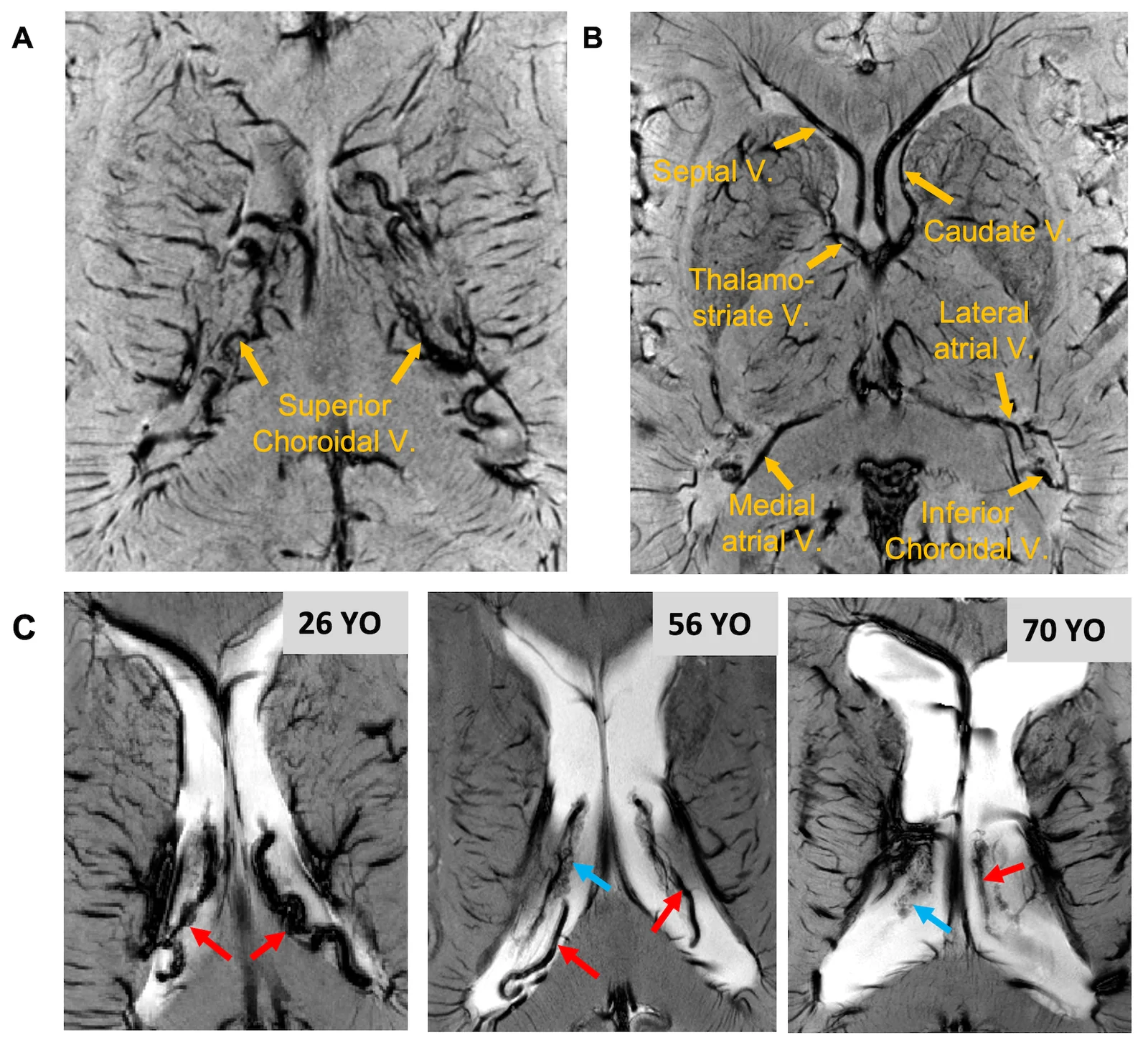
**Visualization of ChP vasculature using gradient echo (GRE) sequences on 7T MRI**. 3D susceptibility-weighted imaging (SWI) revealed (A) superior choroidal vein, and (B) inferior choroidal vein, along with surrounding venous drainage. (**C**) Axial 2D T2*-weighted GRE images from subjects aged 26, 56, and 70 years revealed progressive vessel thinning (red arrows) and an increase in adjacent hypointense, cloud-like structures (blue arrows) with advancing age.

Advanced imaging techniques such as magnetic resonance angiography (MRA) and susceptibility-weighted imaging (SWI) enable visualization of the ChP microvasculature [58], revealing its anatomical features and spatial relationships with adjacent tissues (Fig. 7A-B). Although these methods have limitations in detecting vessels smaller than 500μm and in assessing microvascular blood flow and volume *in vivo*, they still allow the observation of age-related changes, including sparser and thinner vessels (Fig. 7C).

### 3.3 ChP functional and CSF dynamic changes

The aging process impairs ChP function, resulting in reduced CSF production due to diminished blood flow and decreased expression of proteins essential for CSF synthesis [59]. Concurrently, brain tissue volume declines, resulting in a 25-30% increase in ventricular size often seen after the fifth decade of life. The ventricular system expansion prolongs CSF turnover time [60], decreasing from 3-4 times daily in younger individuals to about twice daily in older adults [61]. The slowdown is attributed to increased resistance in CSF drainage pathways, such as arachnoid fibrosis and elevated central venous pressure [62, 63]. Dynamic imaging studies in aged rodent models have shown a significant decline in lymphatic outflow, the primary route for CSF drainage [64] and reduced exchange between CSF and ISF. This is associated with decreased arterial pulsatility and loss of perivascular AQP4 polarization along the penetrating arteries [65], leading to impaired clearance of toxic interstitial solutes and cognitive decline in the elderly [49]. Phase-contrast MRI (PC-MRI) studies have observed reduced CSF flow in elderly patients with cognitive deficits, indirectly indicating ChP dysfunction [66].

With aging, the BCSFB becomes more permeable due to morphological changes in ChP epithelial cells and chronic low-grade inflammation [67]. This increased permeability allows molecules such as albumin to leak from the bloodstream into the CSF. The CSF/serum albumin ratio, commonly measured in the CSF via lumbar puncture, serves as an indicator of BCSFB integrity. Notably, this ratio significantly increases beginning around age 45, suggesting a decline in barrier function [68, 69].

Assessing BCSFB functionality in vivo remains challenging due to its deep anatomical location. However, recent advancements in diffusion-weighted imaging (DWI) and arterial spin labeling (ASL) techniques have facilitated indirect evaluations. For instance, increased apparent diffusion coefficient (ADC) values in the ChP likely reflect age-related microstructural changes [67]. Additionally, ASL-based techniques in animal models have demonstrated a 36% decrease in BCSFB-mediated labeled blood water in aged mice [70]. These imaging biomarkers offer potential for early prediction of neurodegenerative outcomes.

**Figure 8.**
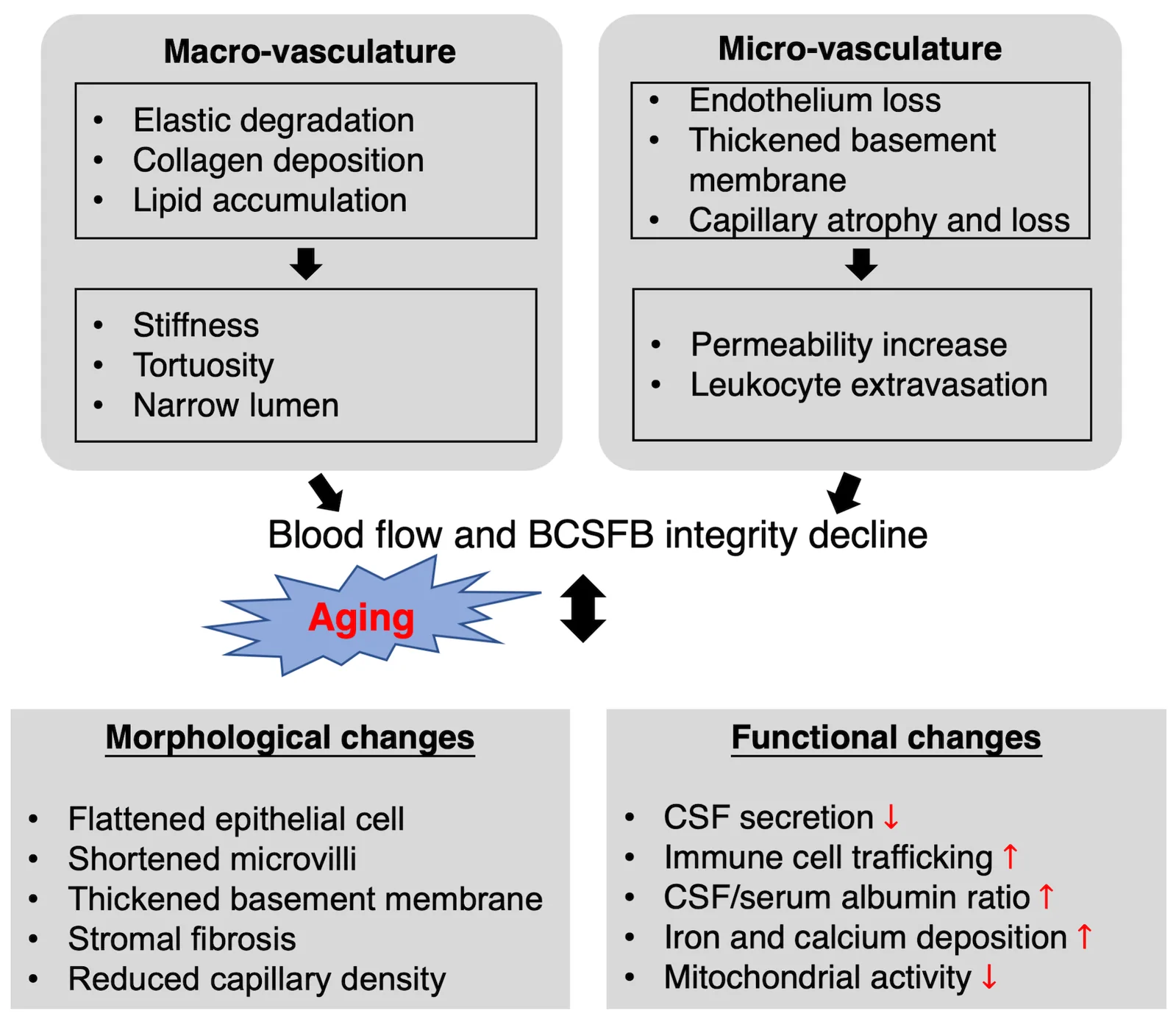
**Overview of age-related changes and their interactions**. The vasculature of the ChP can influence blood flow and the integrity of the blood-cerebrospinal fluid barrier (BCSFB). As a result, the secretion of CSF, the exchange between CSF and ISF, and the molecular composition of CSF could be affected. These changes form the basis of impaired ChP functions, particularly in relation to waste filtration.

The interactions between vascular degeneration in the ChP and morphological or functional changes associated with aging are summarized in Figure 8.

### 3.4 Immune response alterations

As an individual age, immune cell infiltration into the CNS increases, likely due to BCSFB dysfunction [71]. T-lymphocytes and neurotrophic factors that are associated with CNS integrity and functions like hippocampal neurogenesis and spatial learning, accumulate in the ChP stroma and on the apical surface of its epithelium [72, 73]. Baruch et al. reported that CNS-specific T cells modulate brain function via the ChP, and that aging may shift the immune environment toward a Th2-like pro-inflammatory state—characterized by reduced interferon-gamma (IFN-γ) and elevated interleukin-4 (IL-4)—which can negatively impact brain function [74]. Increasing evidence suggests that age-related decline in immune function, referred to as "inflammaging", contributes to the pathogenesis of AD [75, 76]. T cell infiltration is more pronounced in AD brains compared to other non-AD dementias, with especially high concentrations in the hippocampus and temporal cortex [77]. Notably, structural degradation of the ChP may accompany AD pathology and neuroinflammation even before the onset of clinically detectable cognitive symptoms [78]. In addition, genome-wide association studies have identified multiple immune-related gene variants linked to neurodegenerative diseases such as Parkinson’s disease and amyotrophic lateral sclerosis [79]. The activated peripheral immune cells reside at the ChP, surveil the CNS and transport the antigens from the CNS to the deep cervical lymph nodes via the drainage of CSF [80]. Although direct imaging evidence on immune responses are limited, MRI studies have shown ChP enlargement in various conditions, which may indicate that increased immune cell infiltration and interaction with ChP tissue are common pathways in various neurodegenerative diseases.

### 3.5 Calcification and iron deposition

**Figure 9.**
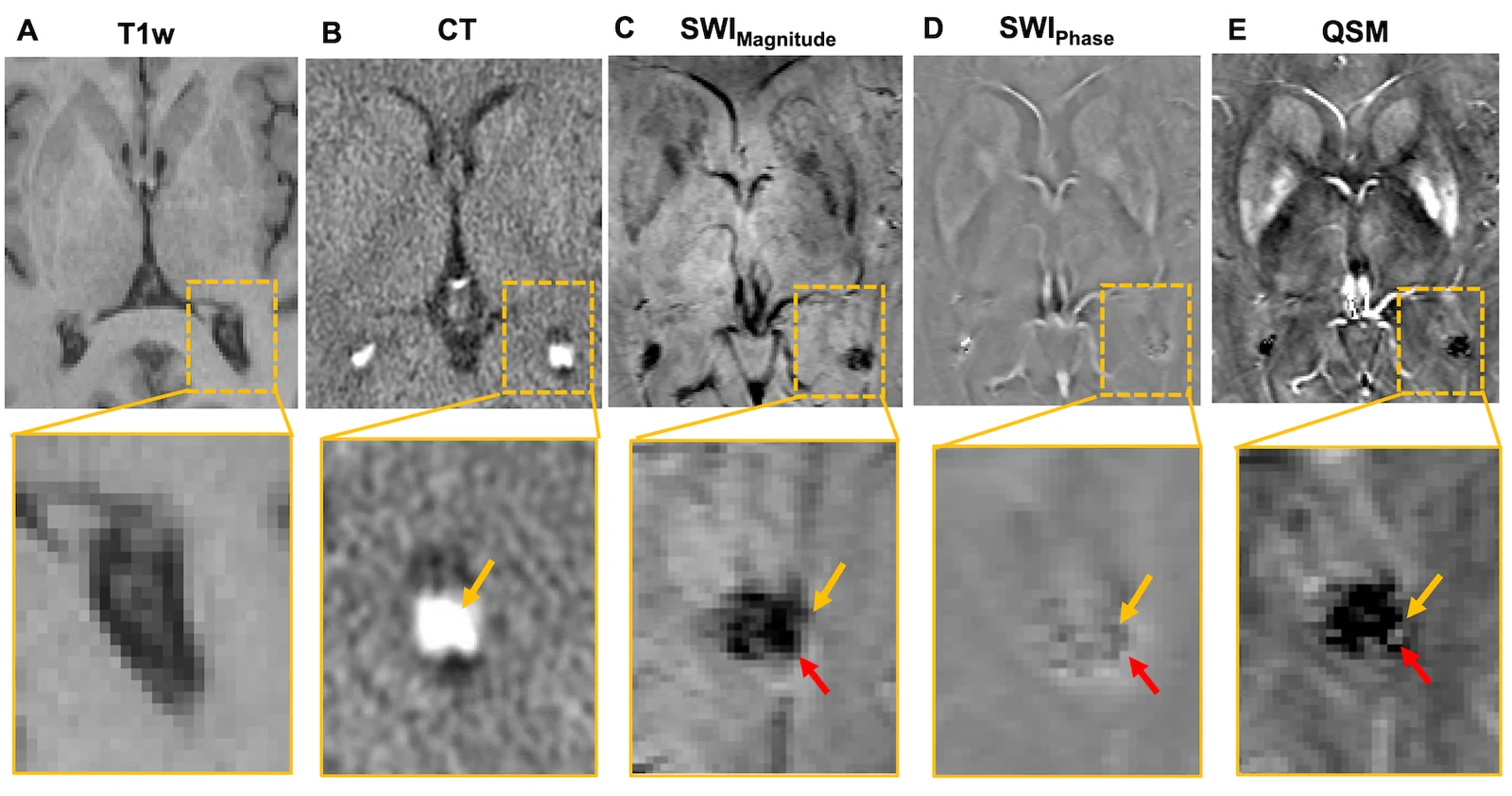
**Imaging findings of the dystrophic calcification in the ChP**. (**A**) T1-weighted MRI does not clearly depict the calcification. (**B**) Computed tomography (CT) reveals hyperattenuating calcifications (yellow arrow), typically interpreted as physiologic change. (**C**) On the SWI magnitude image, both venous blood (red arrow) and calcification (yellow arrow) appear hypointense; (D) However, on the SWI filtered phase image, calcification (diamagnetic) appears hyperintense, whereas veins (paramagnetic) appear hypointense. (**E**) On QSM, the ChP calcification (yellow arrow) appears hypointense, while venous blood (red arrow) appears hyperintense.

The ChP calcification is commonly considered a benign and physiological aspect of aging with prevalence increasing from 1% in the first decade to 19% by the eighth decade [81, 82]. Age-related endothelial cell damage in capillaries and arterioles is hypothesized to increase vascular permeability, facilitating the leakage and deposition of electron-dense psammoma bodies in the capillary walls, thus leading to dystrophic deposits of calcification [83]. Post-mortem examinations using stains such as Mallory have shown that ChP concrements are primarily composed of hydroxyapatite (Ca_10_(PO4)_6_ (OH)_2_) with a low degree of crystallinity, whereas those found in the pineal gland are predominantly made of highly crystalline hydroxyapatite [84]. ChP concrements typically present as either layered calcifications or calcified cores. Compared to conventional MRI sequences, CT reveals up to 15 times more calcifications, with an incidence rate reaching 86% in individuals in their eighth decade of life (Fig. 9A-B) [85]. Advanced MRI techniques, such as SWI and processed quantitative susceptibility mapping (QSM), enhance the detection of calcifications by leveraging their diamagnetic properties, thereby enabling differentiation from paramagnetic substances such as venous blood [86] (Fig. 9C-E). While often benign, ChP calcifications have been associated with various intracranial pathologies, including trauma, metabolic disorders, cerebrovascular diseases, and tumors [87]. Additionally, concurrent calcifications in the ChP and pineal gland may disrupt melatonin secretion, potentially affecting circadian rhythms (e.g. sleep) and the glymphatic system [88, 89].

Beyond calcium, iron accumulation in the ChP is another age-related change [90]. Although iron is essential for brain function, excessive amounts can promote oxidative stress and contribute to neurodegenerative diseases like AD [91]. Aging-related oxidative stress and compromised BCSFB integrity may exacerbate iron deposition [92]. Aged ChP epithelial cells may modify the expression of iron-carrier proteins and their receptors, thereby affecting iron homeostasis [93]. Numerous studies have explored age-related iron deposition in regions critical to neurodegenerative diseases, such as the thalamus, caudate, palladium, hippocampus, amygdala, and nucleus accumbens, indicating an accumulation of non-agglomerated tissue iron [94]. Imaging studies using T2*-weighted MRI have identified hypointense signals in the ChP indicative of iron accumulation, findings that have been corroborated by histological analyses [95]. Furthermore, it was established that the volume of hypointense signal on T2*-weighted imaging and hyperintense signal on QSM, indicative of iron not calcium, was associated with age and plasma Aβ1-42 levels [96].

### 3.6 Proteomics of ChP in aging and dementia

Proteomics is being used to define molecular mechanisms associated with disease pathology and findings on imaging, as well as identify novel biomarkers and therapeutic targets. Transcriptomics may be complementary but shows a poor correlation to proteomics analyses [97, 98]. There are very limited proteomic studies of the ChP in AD, ADRD, and aging. A previous proteomic study with limited detection techniques evaluated the ChP across the Braak tau pathology spectrum and identified several altered proteins, including 14-3-3 proteins [99]. We recently performed a proteomic analysis of the microdissected ChP adjacent to the hippocampus at the level of the lateral geniculate nucleus from autopsy tissue in severe AD cases [100]. When comparing AD and control cases, 616 proteins were differentially abundant and the top signaling pathways associated with the altered proteins included most significantly activated fatty acid beta-oxidation and inhibited glycolysis indicating a shift in cell energy metabolism. Another study evaluated the lateral ventricle walls, including ependymal cells, from nonagenarian, AD, and control cases [101]. When comparing nonagenarians and control cases, most significantly there was altered phagosome maturation, impaired tight-junction signaling, and glucose/mannose metabolism; in AD vs control altered mitochondrial bioenergetics, oxidative stress, remodeling of epithelia adherens junction, macrophage recruitment and phagocytosis, and cytoskeletal dynamics; and in AD vs nonagenarians altered oxidative stress, changes in gluconeogenesis-glycolysis pathways, and cellular effects of choroidal smooth muscle cell vasodilation [101]. As noted above, more proteomic studies have been conducted in the CSF, reflecting the combined protein changes from the ChP and other brain regions. CSF protein changes indicate altered astrocyte/microglial and sugar metabolism [102], neuroinflammation, cerebrovascular dysfunction, apoptosis in AD [103], and are reflected in emerging comparisons to histology and MRI [104]. More proteomic studies are needed to better characterize molecular mechanisms in the ChP, across the neuropathology spectrum, by disease progression (i.e. mild cognitive impairment (MCI)), in association with other variables like *APOE* genotype carrier status, sex, and specific cell type changes.

## 4. ChP changes in age-related neurodegenerative diseases

### 4.1 AD and AD-related dementia (ADRD)

AD is characterized by the presence of Aβ plaques and tau deposits, which have been linked to impaired toxic clearance mechanisms [105]. Histologically, the ChP in AD undergoes morphological changes that are similar to normal aging, including epithelial atrophy, basement membrane thickening, stroma fibrosis, and vessel narrowing [5, 60].

Structural MRI studies have shown an increase in ChP volume among AD subjects, with ChP volume correlating with the AD clinical spectrum, from healthy aging to early MCI, late MCI, and AD [8]. Some researchers hypothesized that the mechanism of ChP enlargement is CSF overproduction and providing more CSF-blood protein transporters on the ChP epithelial tissue, thereby enhancing the activity of CSF-dependent brain clearance systems [106]. However, other studies suggested that the CSF production rate significantly reduced in AD [107, 108]. Iliff et al. utilized two-photon imaging in a rodent model, revealing that arterial pulsatility and AQP4 expression may influence the exchange between CSF and ISF through the glymphatic system, facilitating waste clearance via perivascular drainage [35]. This highlights the potential significance of CSF production and dynamics in driving fluid movement within the brain.

Direct imaging of waste clearance in the perivascular spaces in humans presents significant technical challenges. Advanced MRI techniques have been used to assess CSF dynamics in neurodegenerative diseases, revealing reduced CSF flow velocities in the Sylvian aqueduct and cervical subarachnoid spaces in AD patients, which correlate with cognitive decline [66]. A recent study using ASL method with ultra-long echo time to measure water delivery from arterial blood to ventricular CSF suggests that BCSFB dysfunction may precede brain tissue changes in aged mice [70]. As aging is the primary risk factor of dementia, the age-related changes in ChP perfusion and BCSFB function may serve as early indicators of neurodegenerative processes.

In addition to ChP enlargement that widely observed from structural MRI, a multi-model MRI study of cognitively impaired patients revealed decreased ChP permeability, as indicated by lower values of volume transfer constant (K^trans^) and fractional plasma volume (V_p_) derived from dynamic contrast-enhanced (DCE) imaging [8]. This decreased permeability, which likely results from stromal fibrosis, calcification, and vascular thickening, exhibited a negative correlation with ChP volume. These alterations could adversely impact CSF production, circulation, and waste clearance [5, 107, 109]. Moreover, diffusion MRI studies [53, 67] have reported higher ADC values in the ChP of aging and dementia populations, suggesting impaired microstructural integrity and increased BCSFB permeability.

### 4.2 Parkinson’s disease

Parkinson’s disease (PD) is a progressive neuro-degenerative disorder characterized by the aggregation of α-synuclein (α-syn), which is a major component of the pathognomonic Lewy bodies found intracellularly in neurons [110]. Similar to AD, where alterations in CSF drainage and ChP transporter function contribute to pathology by impairing waste clearance, comparable mechanisms may also be relevant in PD. Instead of taking up α-syn from the CSF intracellularly, the ChP may have an inflammatory response to the α-syn pre-formed fibrils (PFF), which further increase the BCSFB permeability [111]. Structural MRI have revealed ChP enlargement in PD patients, similar to findings in AD and ADRD. Larger ChP volume showed negative association with lower CSF α-syn level for two possible reasons: [106] In early-stage PD patients, an increase in ChP volume was associated with a decrease in striatal dopamine transporter availability, more severe baseline motor deficits, a more rapid increase in dopaminergic medication overtime, as well as a higher risk of developing freezing of gait [112]. Multi-shell diffusion MRI was used to investigate the CSF motion at the level of the suprasellar cistern in PD patients, revealing a reduction in CSF motion compared to age-matched healthy participants. This reduced motion was found to be inversely associated with ChP perfusion [113]. These findings indicate that ChP may upregulate CSF production in response to circulating CSF markers, in the presence of reduced intracranial CSF motion. This perspective appears to contradict the notion that decreased ChP perfusion leads to a decline in CSF production, which is often observed in normal aging [114]. Further research is needed to fully understand this complex relationship.

### 4.3 Amyotrophic lateral sclerosis

Amyotrophic lateral sclerosis (ALS) is a fatal neurodegenerative disease that typically leads to death within 2-5 years of diagnosis. Although ALS can occur at any age, symptoms most commonly develop between 55 and 75 years of age, with aging being the primary risk factor. The pathological mechanisms underlying ALS remain poorly understood. Recent structural MRI studies have revealed ChP enlargement along with impaired BCSFB in ALS subjects [115]. As in other neurodegenerative diseases, ChP abnormalities may contribute to ALS progression by promoting the accumulation of neurotoxic proteins in the CSF, driven by increased vascular leakage and reduced clearance [115-117]. Overall, ChP abnormalities and neurotoxic protein accumulation associated with dysfunctions of BCSFB, and waste clearance may be common features in neurodegenerative diseases.

### 4.4 Idiopathic normal pressure hydrocephalus

Idiopathic normal pressure hydrocephalus (iNPH) is a neurological disorder usually diagnosed in the elderly, characterized by enlarged cerebral ventricles and fluctuating intracranial pressure. Research shows that the prevalence of AD pathology is higher in iNPH patients with more severe dementia [118, 119].

The precise mechanisms governing CSF secretion, flow, and reabsorption in iNPH remain unclear. It has been revealed that the reflux flow from the third ventricle into the lateral ventricle is restricted in iNPH [120]. GBCA-enhanced MRI studies have shown altered intracranial kinetics and decreased molecular clearance from the ChP in iNPH patients [121, 122]. Aging leads to decreased intracranial compliance and diminished arterial pulsatility, which slow down glymphatic flow and convective solute transport. The relative reduction in the ISF coupled with low tissue elasticity in the periventricular area could produce ventricular enlargement [123]. These factors may explain the high overlap between iNPH and AD, highlighting the critical role of CSF dynamics and glymphatic function in the development and progression of both conditions.

### 4.5 ChP and xanthogranuloma

Small ChP cysts, often incidentally discovered in the atrium of the lateral ventricles during postmortem examination, are typically less than 1 cm in diameter. They are lined by compressed connective tissue or epithelium and do not cause signs or symptoms of CSF obstruction [81, 124]. These cysts are more prevalent in older age groups, suggesting a likely degenerative origin. They contain fluid resembling CSF but with a higher protein concentration than CSF [125]. In elderly individuals, these cysts often appear hyperintense on diffusion MRI, likely due to gelatinous cystic changes associated with aging. Studies using oscillating gradient spin-echo (OGSE) sequences with short diffusion times have shown that ChP cysts exhibit lower ADC values than CSF, indicating restricted diffusion and increased viscosity within the cysts [125, 126].

## 5. Imaging techniques for detecting alterations in the choroid plexus

Given the ChP's deep location within the brain’s ventricular system, imaging techniques have emerged as crucial tools for the in vivo assessment of its structural and functional characteristics. The morphology of the ChP is typically evaluated with CT scans and structural MRI. Advanced MRI techniques, along with positron emission tomography (PET), offer avenues for assessing ChP functionality, including perfusion and permeability. Multiple imaging studies with different techniques have indicated that changes in ChP volume, perfusion, permeability, glucose metabolism, and inflammatory activity could potentially serve as biomarkers for ChP pathologies associated with aging process and neurological disorders [8, 53, 127, 128]. A summary of the various applications of different imaging methods is presented in Table 1.

**Table 1:** Summary of imaging techniques for the choroid plexus morphology and functionality.

Study	Objects	Imaging sequences	Measurements	Findings
***ChP Volume***				

Choi et al. (2022) ^[8]^	532 subjects including 78 SCI, 158 early MCI, 149 late MCI, and 147 AD.	3T; T1-weighted; DCE-MRI, QSM	ChP volume; Permeability (K^trans^, V*_p_*); Susceptibility	1) The enlarged ChP volume was associated with the cognitive impairment severity; 2) ChP permeability was negatively correlated with ChP volume; 3) The ChP susceptibility did not show significant difference among groups.
Alisch et al. (2021) ^[53]^	155 subjects with normal cognition from lifespan	3T; 3D T1-weighted SPGR, 3D T2-weighted bSSFP, DWI, pCASL	ChP volume; Relaxometry values (T1 and T2 value); Microstructural integrity (FA, MD); Perfusion (CBF)	1) ChP volume increased with age; 2) T1, T2 and MD nonlinearly increased with aging, whereas FA and CBF nonlinearly decreased with aging; 3) T1, T2, and MD decreased with CBF, whereas FA increased with CBF.

***ChP Perfusion and BCSFB Function***				

Bouzerar et al. (2013) ^[129]^	15 patients with small cerebral lesions	3T; DSC, T1, T2*, FLAIR, T2 FSE	Perfusion (rCBV, rCBF, MTT, SSD, K_2_)	1) K_2_ significantly decreased with aging, whereas MTT significantly increased with aging; 3) No significant correlation was found for age related changes in rCBV and rCBF.
Nathoo et al. (2016) ^[130]^	Male rats; 2 cold injury model, 5 LPS-associated inflammatory model, 7 hypoxia model	9.4T; Pre- and post-contrast T1-weighted (0.5 mmol/kg gadodiamide)	BCSFB integrity (Pre-/post-Gd signal ratio)	Enhanced average intensity in the ventricles and periventricular structures were observed in the inflammatory and hypoxia model, indicating BCSFB impairment.
Anderson et al. (2022) ^[131]^	11 cognitive impaired subjects, 28 cognitively normal subjects.	7T; DCE-MRI, fast 3D GRE (FLASH) sequence (0.1 mmol/kg gadoteridol)	BCSFB integrity (K^trans^, K_co_)	1) K_co_ and K^trans^ declined with age; 2) K_co_ was associated with cognitive dysfunction, indicating the metabolic disturbances and energetic compromise at the BCSFB.
Evans et al. (2020) ^[70]^	12 aged mice (23 months) and 12 adult mice (6 months).	9.4T; ASL-based sequence with ultra-long echo time	ChP perfusion (blood flow), BCSFB function	The aged cohort had a 13% reduction in cortical perfusion and a 36% reduction in rates of BCSFB-mediated blood water delivery to ventricle, suggesting BCSFB function may be more vulnerable to aging.
Zhao et al. (2020) ^[23]^	7 healthy young adults	3T; T1-weighted, stack-of-spiral FSE pCASL	ChP perfusion (apparent blood flow)	1) The ChP has a significantly longer T1 than GM; 2) ChP blood flow was 1.6 times higher than the GM, with shorter ATT (not statistically significant).

***ChP Microstructure***				

Alicioglu et al. (2017) ^[67]^	202 healthy subjects with wide age range.	1.5T; DWI	ChP microstructure (ADC)	1) The ADC value increased significantly after the age of 61 years old; 2) Female had a more significant increase of ADC compared to male.
Maekawa et al. (2019) ^[126]^	27 subjects with wide age range (22-89 years old).	3T; OGSE DWI sequence.	ChP microstructure (ADC)	The ChP cysts had lower ADC values in comparison with surrounding CSF, indicating restricted diffusion property and higher viscosity.

***ChP Calcium and Iron Deposition***				

Chen et al. (2014) ^[132]^	Human; 38 patients with intracranial calcifications and/or hemorrhages.	GRE-SWI and QSM	ChP calcification	QSM is superior to GRE pahse imaging in the differentiation of intracranial calcifications from hemorrhages with regard to the sensitivity and specificity.
Yalcin et al. (2016) ^[47]^	Human; 11941 subjects with age ranging from 15 to 85 years old.	CT	ChP calcification	1) 70.2% subjects had ChP calcifications with male dominance; 2) ChP calcifications are frequently observed in the elderly population.
Joseph-Mathurin et al. (2013) ^[96]^	Animals; 20 mouse lemurs aged 4.1 to 6.4 years.	7T; T2 and T2*-weighted.	ChP iron deposition	Quantification revealed an increased size of hypointense signals, corresponding to the iron deposition, in animals treated with Aβ_1-42_.

**Note:** SCI (subjective cognitive impairment); MCI (mild cognitive impairment); SPGR (spoiled gradient recalled echo); bSSFP (balanced steady state free precession); rCBV (relative cerebral blood volume); rCBF (relative cerebral blood flow); MTT (mean transit time); SSD (signal slope decrease); K_2_ (permeability parameter); LPS (lipopolysaccharide); K^trans^ (contrast agent extravasation rate); K_co_ (water efflux rate from choroid plexus to the ventricle); V*_p_* (fractional plasma volume); ATT (arterial transit time); OGSE (oscillating gradient spin-echo); TSPO (translocator protein).

### 5.1 MRI

#### 5.1.1 Structural MRI

**Figure 10.**
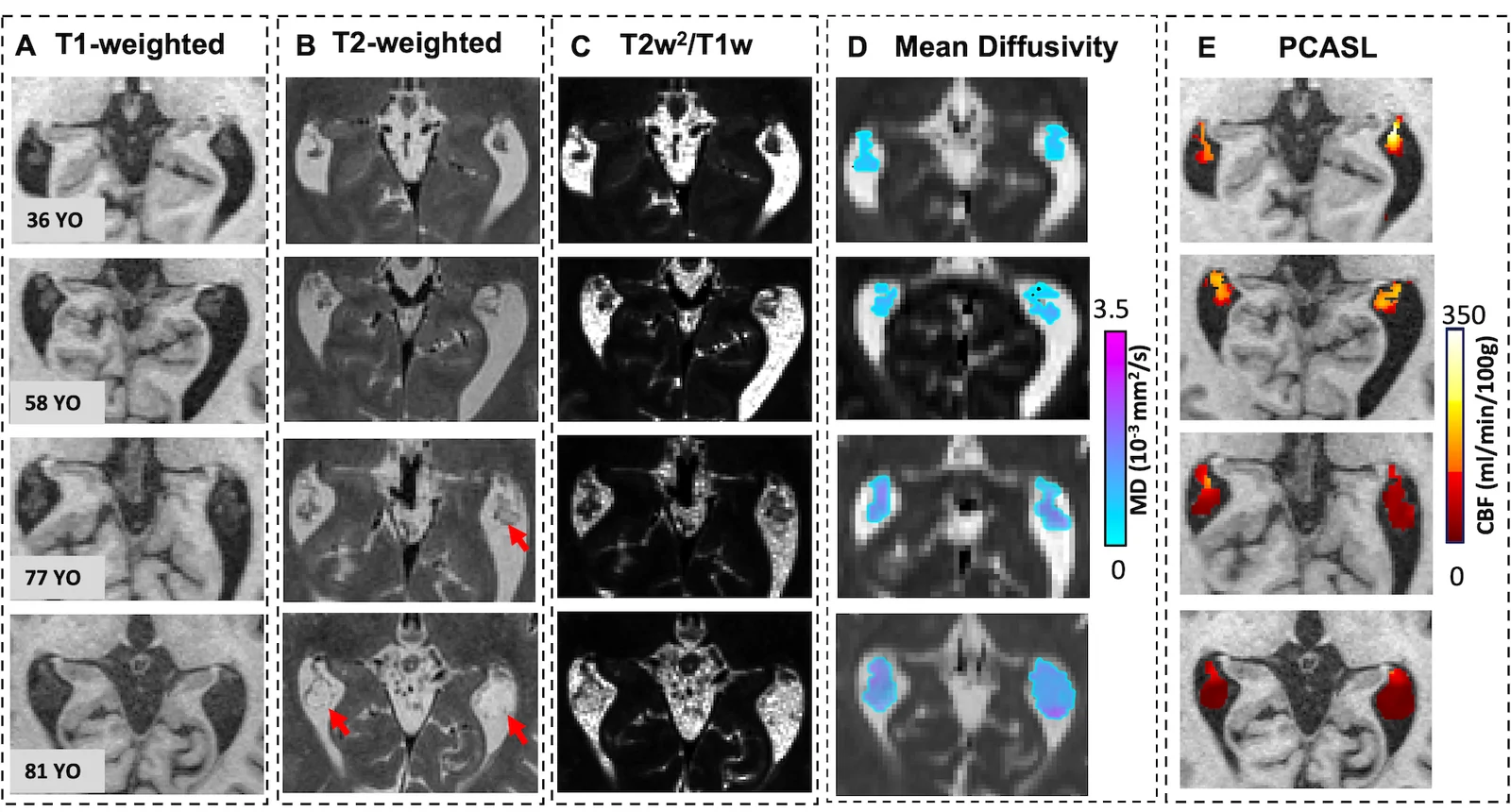
**Age-related alterations of the choroid plexus in different MRI modalities**. (**A**) With aging, the ChP volume is getting large on T1-weighted MRI without contrast agent. (**B**) In T2-weighted MRI, the ChP is hypointense and becomes less dense with aging. (**C**) The T2w2/T1w ratio mapping enhances the contrast between vessel and non-vessel compartment of ChP; (D) The outline of ChP can be better delineated in the mean diffusivity (MD) map, where MD values increase with age within the ChP. (**E**) The CBF of the ChP is lower in the aged population than young population. Adapted from Ref [114].

Structural MRI, particularly T1w MRI, facilitates the evaluation of the ChP by assessing its signal, size, and shape. Numerous studies have consistently reported ChP enlargement associated with normal aging and various neurological diseases [8, 53, 133]. ChP volume can serve as an independent imaging marker for cognitive function [8]. However, accurate automatic delineation of the ChP remains challenging due to limited imaging resolution, tissue contrast, and partial volume effects (PVEs). Currently, manual segmentation remains the gold standard. It becomes increasingly challenging when age-related stromal changes alter the tissue appearance and contrast (Fig. 10A). Although GBCAs can enhance ChP visibility on T1w MRI, their routine use is often restricted in certain clinical and research settings. On T2-weighted (T2w) MRI, the ChP typically shows signal dropout or flow void (dark signal) due to the rapid outflow in the highly vascularized glomus. For example, the last image of Figure 10B, from an 81-year-old subject, displays sparse hypointense vessels encompassing a cyst-like structure. Combining T1w and T2w images may improve tissue contrast and image homogeneity [134]; for instance, as shown in Figure 10C, the T2w^2^/T1w fusion image enhances signal dropout and tissue contrast, leading to darker vessels, brighter CSF, and less intense appearance of the cyst [114].

Recent advances in automatic segmentation algorithms, such as Bayesian Gaussian Mixture Models (bGMM) and 3D U-Net convolutional neural networks, have significantly improved the accuracy of ChP segmentation on non-contrast T1w MRI [135, 136]. This is crucial for subsequent functional assessments related to aging and dementia, including analyses of microstructure and hemodynamics (Fig. 10D-E). Refined in vivo imaging techniques with higher resolution and tissue contrast, along with robust segmentation algorithms, are necessary for future investigations into ChP morphology. For instance, T2-weighted imaging using ultra-high field 7T MRI enables detailed visualization of ChP structures, including the improved detection of vasculature and cyst formations. In a recent study, a higher prevalence of ChP cysts was observed in patients with AD, suggesting a potential link between AD pathology and cyst formation [137]. This association may be mediated by the accumulation of Aβ and tau proteins, which can further induce cellular apoptosis and inflammatory responses within the ChP.

#### 5.1.2 Vascular MRI

The ChP contains dense networks that are essential for CSF production and maintaining brain homeostasis. Investigating its vascular anatomy and function is essential for understanding its involvement in neurological disorders. In the clinical neuroimaging research, the following vascular MRI techniques, with or without contrast agents, are commonly employed in studying the alteration of ChP’s intricate vascular networks.

*Dynamic susceptibility contrast (DSC) MRI:* DSC-MRI is a widely used perfusion imaging technique, aiding in the diagnosis and monitoring of various neurological conditions such as brain tumors, ischemic stroke, vascular malformations, and neurodegenerative diseases. It utilizes rapid T2*-weighted echo-planar imaging (EPI) sequences acquired within approximately 60 seconds following the contrast agent administration. By measuring susceptibility-induced signal changes as the contrast passes through cerebral vessels, DSC-MRI allows for the creation of perfusion maps, including relative cerebral blood volume (rCBV), relative cerebral blood flow (rCBF), mean transit time (MTT), and capillary permeability (*K*_2_) [138, 139]. Bouzerar et al. utilized DSC-MRI to evaluate age-related changes in ChP perfusion in patients with brain tumors and found a significant reduction in *K*_2_ with increasing age, while no correlations were observed for rCBV and rCBF changes related to age [129]. The decreased *K*_2_ may reflect structural alterations, such as thickening of the stromal and basement membrane, as previously observed in histological studies of aged rat and sheep models [46, 140]. However, the application of DSC-MRI for permeability assessment is limited by its relatively low spatial resolution and potential confounding effects from leaky ChP capillaries. These limitations necessitate cautious interpretation of DSC-MRI data.

*Dynamic contrast-enhanced (DCE) MRI:* DCE-MRI is a noninvasive technique that assesses blood flow and BBB permeability by tracking T1 signal changes following GBCAs administration. It has proven effective in evaluating conditions such as tumors, MS, strokes, and AD [141-143]. Quantitative analysis involves pharmacokinetic modeling of contrast exchange between the blood plasma and the extravascular extracellular space (EES) [144], commonly characterized by the volume transfer constant (K^trans^). These models rely on an arterial input function (AIF) to determine the concentration of the tracer in the blood plasma over time. Additionally, the initial area under the gadolinium concentration (IAUGC) time curve can be calculated to assess the vascular compartment. Compared to DSC-MRI, DCE-MRI offers higher spatial resolution (e.g., 1×1×1 mm³) but lower temporal resolution (~20 seconds per frame) and generally requires longer scan times (over 15 minutes). Accurate estimation of AIF estimation and appropriate kinetic model selection are critical for reliable tissue permeability measurements.

A modified two-compartment pharmacokinetic exchange model analyzing the longitudinal relaxation rate constant (R_1_) time course has been proposed to determine the water efflux rate constant (K_co_) from the ChP to the ventricle and K^trans^ from plasma (V*_p_*) to interstitium (V*_e_*) [131]. Studies indicated that both K_co_ and K^trans^ decrease with aging, with reduced K_co_ linked to cognitive dysfunction, potentially reflecting metabolic dysfunction at the BCSFB. A decline in K^trans^ in older individuals suggests decreased microvascular permeability, corroborated by the DSC-MRI study [129]. Moreover, in AD, the Patlak model has shown reduced K^trans^ in patients compared to late MCI cases, and lower V*_p_* has been associated with more severe cognitive decline [8].

With a small molecular weight (~600 Da), GBCAs are prone to leakage into the EES, especially given the permeability of the BCSFB. Kao et al. observed the entry of gadolinium into the CSF in the lateral ventricles via the ChP following intravenous GBCA injection [145]. However, variability in DCE-MRI measurements can arise from factors such as AIF determination, contrast agent dose, and kinetic model. While complex models yield detailed permeability insights, they demand precise parameter fitting and higher imaging resolution. Future research should focus on standardizing imaging and analysis protocols of DCE-MRI to improve reliability when evaluating subtle permeability.

*Arterial spin labeling (ASL):* ASL is a non-invasive MRI technique that quantifies CBF by using magnetically labeled arterial blood water as the endogenous contrast agent. However, measuring blood flow within ChP presents challenges, including PVEs from surrounding tissues and limited resolution, which can reduce accuracy in capturing rapid or dynamic perfusion changes within the ChP. To overcome these limitations, a recent human study acquired multiple ASL images with repeated control/label pairs and employed voxel-wise apparent blood flow calculations, allowing for the quantification of ChP perfusion dynamics and spatial distribution [23]. Compared to GM, the ChP exhibited a longer T1 relaxation time, indicating that labeled water transfers from arterial blood to CSF. Additionally, the ChP showed an approximately 1.6 times higher blood flow [23] and shorter arterial transit times (ATTs) [114] than GM, likely reflecting the influence of different feeding arteries. It has been shown that ChP perfusion is directly related to cranial-to-caudal CSF flow through the cerebral aqueduct, as measured by phase-contrast MRI [146]. Thus, ChP perfusion may serve as a proxy for estimating CSF production rate.

Beyond measuring CBF, ASL can also assess CSF movement and BCSFB function. Time-spatial labeling inversion pulse (Time-SLIP), an ASL variant, selectively suppresses CSF signal in a target region using inversion recovery (IR) pulses. This creates contrast for visualizing CSF flow dynamics as non-labeled, high-signal CSF moves into the selected region [147]. This technique visualizes both linear and turbulent CSF flow, aiding in the diagnosis of conditions like hydrocephalus by confirming the presence or absence of CSF movement from tagged regions into adjacent compartments [148]. Here is a trade-off: while more selective labeling is beneficial in complex anatomical regions, it may lead to decreased labeling efficiency, making it challenging to visualize CSF flow dynamics.

Another BCSFB-ASL sequence with ultra-long TE (~220ms) was developed to isolate the CSF signal and quantify water delivery rates from arterial blood to ventricular CSF via the ChP, effectively mitigating PVEs [70]. Using this method, researchers found that the vasculature of the BCSFB in aged mice is more susceptible to vasoconstriction than the vessels in the cortex, indicating that BCSFB dysfunction may serve as a sensitive early biomarker for neurodegenerative processes [70, 149].

#### 5.1.3 Diffusion-weighted (DWI) and diffusion tensor imaging (DTI)

Diffusion-weighted and diffusion tensor imaging (DWI/DTI) are valuable tools for assessing microstructural changes in ChP. DWI measures the apparent diffusion coefficient (ADC), reflecting water molecule mobility [67], with higher values indicating a greater water molecule mobility. Elevated ADC values in older individuals suggest increased extracellular space and potential degradation of the blood-cerebrospinal fluid barrier (BCSFB) integrity[53, 67]. DTI provides additional metrics, such as fractional anisotropy (FA) and mean diffusivity (MD), which can indicate microstructural integrity. Decreased FA values may reflect age-related microstructural impairments in the ChP [53]. A study using both DWI and T1/T2 mapping found that reduced microstructural integrity of the ChP, indicated by higher T1, T2, and MD values, or lower FA values, was associated with poorer cognitive performance, particularly in processing speed and fluency [11], and these microstructural measures were more sensitive to cognitive decline than macrostructural metrics such as ChP volume.

ChP cysts often appear hyperintense on DWI due to restricted diffusion, with lower ADC values compared to CSF, indicating increased viscosity, possibly from cholesterol crystals [126]. Studies have found a positive correlation between age and the ADC value in the ChP [53, 67]. Despite lack of the histopathological validation, the effects of the ChP sub-structural changes on diffusion metrics cannot be disregarded and require further elucidation.

Recently, the DTI analysis along the perivascular space (DTI-ALPS) index has been proposed to assess glymphatic system function by measuring water diffusivity along perivascular spaces [150]. Higher ChP volume has been associated with slower glymphatic clearance rates and increased WMH growth, suggesting that ChP enlargement may impair glymphatic clearance [151]. However, the DTI-ALPS index's reliability is still under investigation due to potential confounding factors and the complexity of accurately measuring glymphatic function [152].

#### 5.1.4 2D and 3D T2*-weighted gradient echo (GRE) on ultra-high field 7T MRI

**Figure 11.**
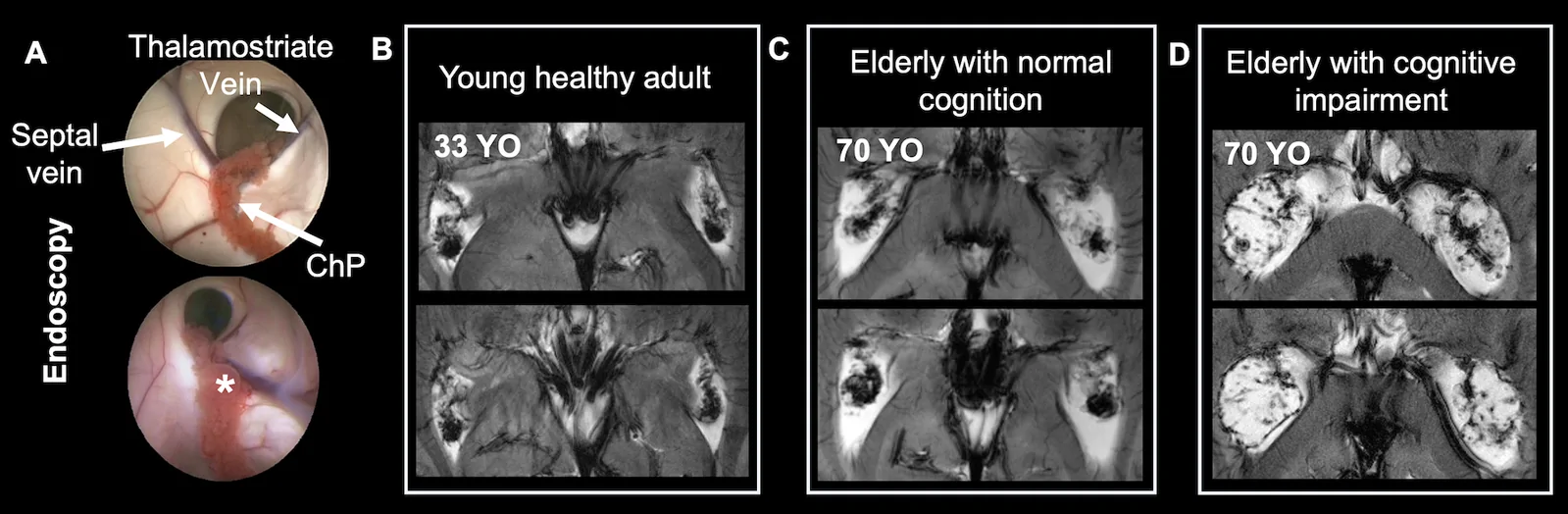
**Ultra-high field (7T) MRI provide details of the ChP sub-structures**. (**A**) Invasive endoscopy images showed ChP glomus with granular appearance tangling with supplying arteries/drainage veins. It also revealed cyst (asterisk) structure of the ChP (Adapted from Ref [153]). ChP images using 2D GRE on 7T MRI of (B) a young healthy adult, (C) an old subject with normal cognition, and (D) an old subject with cognitive impairment, respectively.

Local magnetic field heterogeneity, caused by paramagnetic, diamagnetic, or ferromagnetic substances, leads to T2 shortening and results in signal loss on T2*-weighted GRE sequences. Using high-resolution T2*-weighted 2D GRE on 7T MRI, the ChP glomus have a granular appearance intertwined with arteries and veins, along with small cysts (Fig. 11A) [58], aligning with the endoscopic findings [153]. In a young healthy adult, 2D GRE data from 7T MRI reveal the ChP within the trigone region of the lateral ventricle as densely packed with hypointense vascular structures, surrounded by iso-intense stromal tissue and hyperintense CSF (Fig. 11B). In contrast, an older individual with normal cognition exhibits a loosely packed vasculature with some intact vessels, whereas an older subject with cognitive impairment presents an enlarged ChP with large cysts occupying the trigone region (Fig. 11C-D).

Compared to 3T, 7T MRI offers superb signal-to-nose (SNR) and susceptibility contrast, which allows high-resolution T2*-weighted imaging or SWI, which have higher sensitivity to susceptibility components such as iron, calcium, and deoxygenated hemoglobin [154]. The magnetic susceptibility contrast in SWI is amplified both by the prolonged TE during image acquisition and by applying a post-processing phase mask [155]. QSM, derived from 3D GRE data, provides quantitative assessments of tissue magnetic properties and helps distinguish between calcifications (diamagnetic) and venous blood (paramagnetic) [132, 156, 157], as shown in Figure 9E. QSM has been proposed to outperform GRE phase imaging in differentiating intracranial calcifications from hemorrhages [132]. In support of this, a negative correlation was observed between CT attenuation values and QSM susceptibility values within ChP calcifications [158], suggesting that QSM can accurately measure calcium deposition with reference to CT.

However, studies using 3T MRI have reported inconsistent findings regarding ChP susceptibility changes in conditions such as AD and MCI. For instance, one study found no significant differences in ChP susceptibility among early MCI, late MCI, and AD groups, nor any correlation between susceptibility and ChP volume [8]. In contrast, another investigation—featuring a larger cohort and broader age distribution—reported a negative association between extensive ChP calcification and both regional cortical thickness and subcortical volumes. This study also observed a link between ChP calcification and maternal history of AD or ADRD [159]. The discrepancies among studies may reflect differences in imaging resolution, sample size, and susceptibility artifact correction methods.

**Figure 12.**
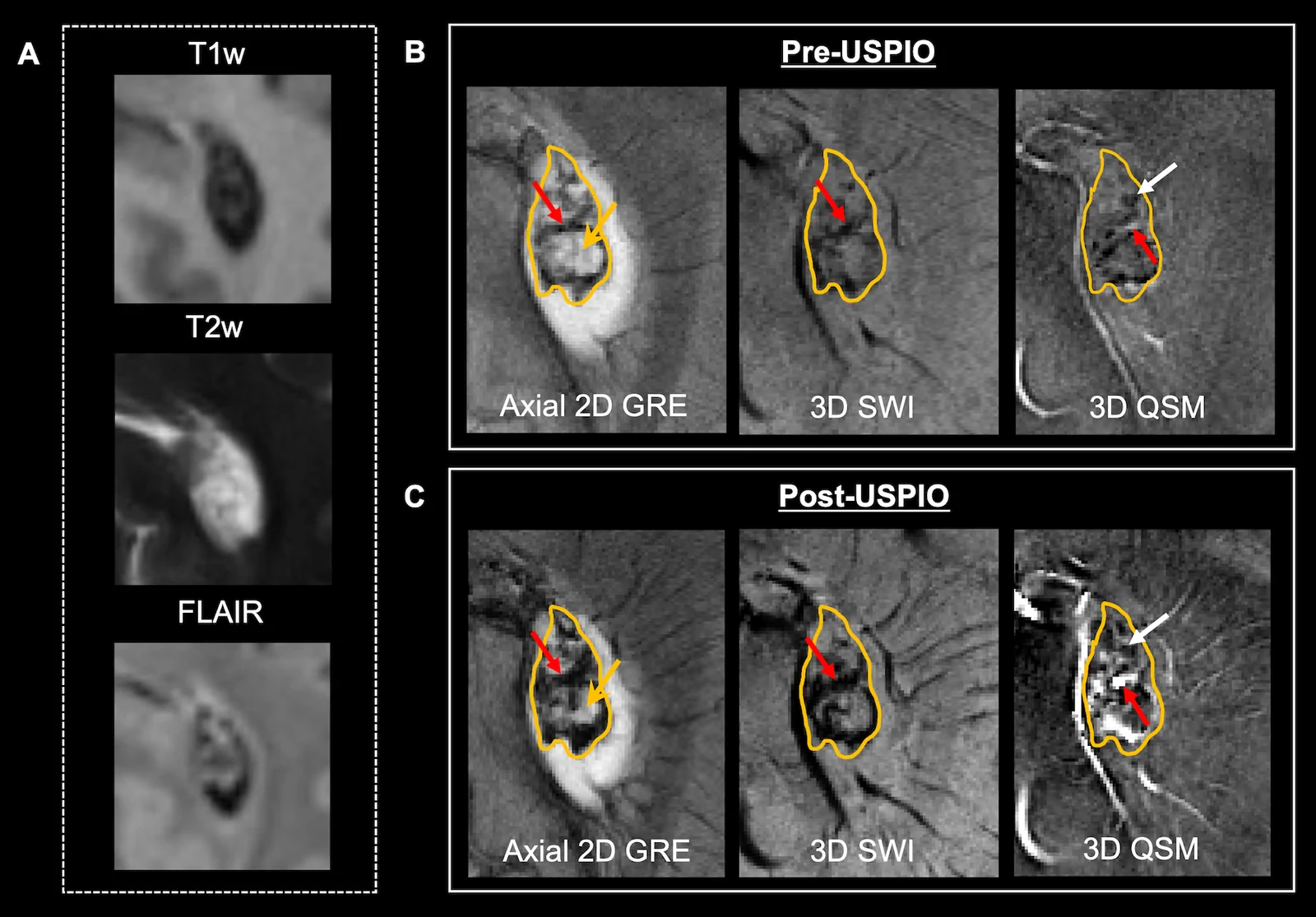
**Contrast enhanced MRI with USPIO**. (**A**) The ChP is not clearly depicted by conventional MR sequences, with obscure boundary between ChP and CSF. (**B**) Without USPIO, the boundary between CSF and ChP is clearly defined (yellow line) using 2D gradient echo on 7T, with hypointense vascular (red arrows), iso-intense stromal (yellow arrows), and hyperintense CSF components. The venous vessels are dark on SWI whereas bright on QSM. (**C**) After the administration of USPIO, both venous and arterial vessels are highlighted. In QSM data, the susceptibility value increases in vessels after contrast administration, whereas the calcium (white arrows) remains dark due to the diamagnetic property.

Ultra-high field 7T MRI, when combined with ultrasmall superparamagnetic iron oxide (USPIO) nanoparticles such as ferumoxytol, leverages the susceptibility-induced blooming effects to achieve high-resolution and high-contrast imaging. Ferumoxytol-enhanced 2D and 3D GRE sequences enable detailed visualization of intracranial microstructures, such as small arteries with diameters as small as 50-200μm [57]. Unlike GBCAs, USPIOs behave differently due to their larger molecular size (~50 nm), resulting in slower extravasation compared to the much smaller (~1 nm) gadolinium chelates. Additionally, USPIOs can be trapped by the circulating macrophages, which then cross the BCSFB in response to inflammation and injury. Because of their prolonged circulation time and more uniform distribution, USPIOs provide better ChP microvasculature compared to GBCAs. Animal studies have shown that intravenously administered USPIOs can accumulate within ChP epithelial cells [160]. Compared to conventional sequences (Fig. 12A), USPIO-enhanced 2D- and 3D-GRE, as well as QSM offer more details of the ChP's substructures, including vessels, stromal tissue, and mixed calcium deposits (Fig. 12B-C). These high-resolution images have also revealed age-related morphological changes, such as reduced vascular density and stromal expansion [58].

### 5.2 Positron Emission Tomography (PET) imaging

PET is also a useful tool in detecting age- and neuroinflammatory changes in the ChP. ^18^F FDG-PET imaging reveals temporoparietal hypometabolism, a hallmark of early AD [161], reflecting neuronal degeneration. Amyloid PET imaging shows diffuse β-amyloid deposition, while tau PET imaging with ^18^F AV-1451 (Flortaucipir) highlights region-specific tau accumulation correlating with cognitive symptoms, CBF, brain atrophy and metabolic decrease [162]. Notably, increased ^18^F AV-1451 retention in the ChP, once considered off-target binding, has been linked to Biondi ring tangles within ChP epithelial cells [163], suggesting potential on-target binding beyond the hippocampus [164].

Additionally, PET imaging targeting the 18 kDa translocator protein (TSPO) offers insights into neuroinflammation. TSPO is overexpressed in activated microglia and macrophages, including those in the ChP stroma. Elevated ^18^F-DPA-714 binding in the ChP has been observed in presymptomatic MS patients, indicating early inflammatory activity [165]. This increase may result from CD163+/TSPO+ macrophages with antigen-presenting functions, contributing to ChP inflammation that precedes parenchymal infiltration and demyelination characteristic of MS. Therefore, PET imaging modalities, including tau and TSPO tracers, can provide complementary information on ChP involvement in neurodegenerative diseases, enhancing our understanding of disease mechanisms and progression.

### 5.3 Computed tomography (CT)

CT remains the clinical gold standard for detecting intracranial calcifications, including those in the ChP, due to its high sensitivity to calcium's photon attenuation properties. However, CT provides limited soft tissue contrast, restricting its ability to characterize tissue changes surrounding the ChP.

Conventional MRI sequences often struggle to reliably detect or quantify calcifications because calcium can appear with variable signal intensities. Emerging techniques like QSM offer promising alternatives. QSM leverages calcium's diamagnetic properties to differentiate it from paramagnetic substances like iron, enabling more precise localization and quantification of calcifications. Studies have demonstrated that QSM can detect calcifications with sensitivity and specificity approaching that of CT, potentially offering a non-ionizing alternative for assessing intracranial calcifications [166]. As imaging technologies advance, integrating QSM into clinical practice could enhance our understanding of ChP pathology and its role in neurodegenerative diseases.

Historically considered a benign, age-related finding, ChP calcification is now being linked to neuro-inflammatory processes and cognitive decline [127, 167]. Recent multimodal imaging studies combining PET, CT, and MRI have shown that ChP calcification correlates with cortical microglial activation and ChP enlargement [128]. These findings suggest that ChP calcification may reflect underlying inflammatory changes, such as fibrous stroma expansion and immune cell infiltration, rather than merely representing a passive, age-associated phenomenon.

## 6. Conclusions and future directions

The ChP functions as a dynamic transport interface essential for maintaining brain homeostasis by regulating the exchange between blood and CSF. With advancing age, the ChP undergoes both structural and functional alterations, which are increasingly implicated in the pathogenesis of neurodegenerative diseases. However, it remains unclear whether ChP dysfunction represents a primary driver of disease pathogenesis or a secondary consequence of ongoing neurodegeneration. Several potential ChP-related mechanisms have been proposed to contribute to neurodegeneration. First, reduced CSF production may impair the clearance of neurotoxic proteins and lead to their aggregation within the ChP, potentially further disrupting CSF production. In addition, reduction in CSF production may impair CSF circulation and hinder the clearance of toxins through the perivascular space in brain tissues. Second, alterations in protective and transporter proteins of the ChP may hinder the clearance of harmful substances from the CNS, exacerbating neurotoxic accumulation. Third, disruption of tight junctions between ChP epithelial cells compromises the integrity of the BCSFB, increasing its permeability and allowing infiltration of inflammatory cells, and potentially promoting chronic neuroinflammation on the cortical surface and meninges.

From an imaging perspective, ChP enlargement is frequently observed in both normal aging and various neurological disorders. This enlargement may reflect stromal thickening, deposition of calcium and lipofuscin, or a compensatory response to the diminished CSF production. Despite inconsistencies across literatures, several advanced imaging studies have reported notable alterations in ChP-associated metrics among aged individuals and patients with AD, including: 1) reduced CBF; 2) microstructural changes, indicated by increased T1, T2, and MD values, which are associated with elevated water mobility and tissue loosening; 3) impaired BCSFB integrity, evidenced by altered pharmacokinetics on contrast-enhanced MRI; 4) reduced net CSF flow through the cerebral aqueduct, suggesting a decline in CSF production and bulk flow. Although the specificity of these alterations to different types of neurodegenerative conditions remains uncertain, they may vary across imaging measures [8]. Moreover, measurements obtained from advanced imaging techniques may be more sensitive to reflect microstructural changes of the ChP, which precedes ChP volume when predicting cognitive decline.

While emerging neuroimaging techniques and proteomic studies [99, 100] have laid the groundwork for mapping ChP degeneration across the lifespan, the studies of underlying mechanisms of ChP-related dysfunctions in CSF dynamics and waste clearance are still at an initial stage of investigation, particularly in AD and ADRD. Age-related changes in the ChP may promote the disruption of glymphatic system and contribute to the cognitive impairment, especially in the presence of additional risk factors such as adiposity, diabetes and other cardiovascular diseases [168]. ChP abnormalities tend to be more pronounced under neurodegenerative or neuroinflammatory conditions, where pathological changes such as neurotoxic protein accumulation and chronic inflammation could further damage both micro- and macrostructural integrity. Therapeutic strategies targeting ChP, such as enhancing regional CBF or modulating immune responses, may offer promising avenues for intervention in AD and other neurodegenerative diseases.

Future studies are needed to correlate histological findings with imaging manifestation, clarify the specific pathological features underlying various neurological and psychological disorders, and link these features to in vivo changes. This will help improve imaging specificity, enabling better disease differentiation and monitor disease progression. Furthermore, advancing imaging biomarkers beyond volume-based metrics could enhance early diagnostic capabilities.
